# Leveraging existing data sets to generate new insights into Alzheimer’s disease biology in specific patient subsets

**DOI:** 10.1038/srep14324

**Published:** 2015-09-23

**Authors:** Kevin D. Fowler, Jason M. Funt, Maxim N. Artyomov, Benjamin Zeskind, Sarah E. Kolitz, Fadi Towfic

**Affiliations:** 1 Immuneering Corporation, Cambridge, Massachusetts, United States of America; 2 Department of Immunology and Pathology, Washington University in St. Louis, St. Louis, Missouri, United States of America

## Abstract

To generate new insights into the biology of Alzheimer’s Disease (AD), we developed methods to combine and reuse a wide variety of existing data sets in new ways. We first identified genes consistently associated with AD in each of four separate expression studies, and confirmed this result using a fifth study. We next developed algorithms to search hundreds of thousands of Gene Expression Omnibus (GEO) data sets, identifying a link between an AD-associated gene (NEUROD6) and gender. We therefore stratified patients by gender along with APOE4 status, and analyzed multiple SNP data sets to identify variants associated with AD. SNPs in either the region of NEUROD6 or SNAP25 were significantly associated with AD, in APOE4+ females and APOE4+ males, respectively. We developed algorithms to search Connectivity Map (CMAP) data for medicines that modulate AD-associated genes, identifying hypotheses that warrant further investigation for treating specific AD patient subsets. In contrast to other methods, this approach focused on integrating multiple gene expression datasets across platforms in order to achieve a robust intersection of disease-affected genes, and then leveraging these results in combination with genetic studies in order to prioritize potential genes for targeted therapy.

In recent years, many investigators have thoughtfully applied genetics and genomics approaches to investigate the biology of Alzheimer’s Disease (AD)^1^,^2^,^3^. These efforts have yielded a rich collection of gene expression and single-nucleotide polymorphism (SNP) data sets, along with extensive analyses of particular data sets. The availability of such studies provides the opportunity to generate fresh insights into the biology of AD, independently of prevailing hypotheses, by integrating existing data sets in novel and innovative ways.

A number of studies have examined ways to do this. For example, Krauthammer *et al*. used seed genes of known importance in AD to identify additional candidate genes using genetic linkage and text-mined protein-protein interaction data via a graph-theoretic method^4^. Chen *et al*. reported a method to rank AD-related genes by importance based on database protein-protein interaction data^5^. Liu and colleagues interpreted genomic and proteomic data using a Bayesian statistical framework with the aim of prioritizing candidate genes, and found that this approach was able to identify known candidate genes for Alzheimer’s^6^. Soler-López *et al*. utilized an initial list of known AD genes to identify additional genes of interest based on combining protein-protein interaction data with criteria of AD- associated genomic locations or changes in gene expression^7^. Caberlotto *et al*. obtained a list of seed genes from a gene expression dataset, SNP data, as well as genes previously identified as potential AD drug targets, and used database protein-protein interaction data to investigate potential underlying biology represented among these genes^8^.

These methods all utilized protein-protein interaction data to help predict which genes might be important in disease. By contrast, the approach reported here uses intersections across multiple datasets to filter for more robust candidate genes, obtaining an intersection of genes from expression datasets that enables targeted mining of SNP data to identify those candidate genes more likely to be causal.

Methodologies for combining heterogeneous data sets to identify robust biological signals are not well established, particularly in the area of integrating gene expression and SNP data from separate cohorts. Differentially expressed genes alone are of limited utility, since they include both downstream signals resulting from disease pathology and upstream signals that may be more causative. Incorporating SNP signals into an analysis can help identify causative signals that may represent more direct targets for new medicines.

In conducting such integrative analyses, it is also critical to consider patient stratification. Today, studies of many different diseases are increasingly finding subsets of patients with distinct patterns of biology^9^,^10^. It is plausible that not all AD patients have identical mechanisms driving their common symptoms and manifestations of the disease. To the extent that AD patients may differ in certain aspects of the biology underlying their disease, it stands to reason that certain medicines may be more, or less, effective in particular subsets of patients.

Therefore, we sought to integrate publicly available gene expression and SNP data sets as a means to generate new insights into the biology of AD, stratify these patients, and generate hypotheses for new subset-specific medicines.

## Results

### Differential expression analysis between AD patients and healthy controls

**Figure 1:**
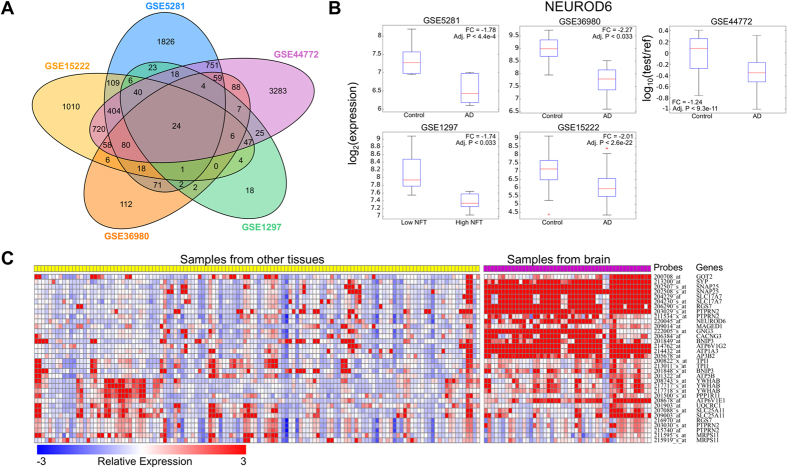
Genes downregulated in AD consistently across 5 data sets. (**a**) Venn diagram illustrating the intersection of significantly downregulated genes across multiple data sets. (**b**) Box plots of NEUROD6 expression in each of the 5 data sets. (**c**) Heat map of the 24 consistently downregulated genes showing specificity for brain tissue.

We first performed an integrated analysis of existing Alzheimer’s gene expression data sets. We identified genes with significantly differing expression levels between healthy controls and AD patients (as defined by overall diagnosis or NFT score) in each of four data sets. Taking the intersection, 25 genes were downregulated significantly with disease in all four data sets. We subsequently obtained a fifth data set (Zhang *et al*.), and observed that in this study 24 out of these 25 genes were also downregulated significantly with disease. We thus identified 24 genes that were significantly downregulated with disease in each of 5 data sets (Fig. 1a and Table 1). Box plots of NEUROD6 (Fig. 1b) and SNAP25 (Supplementary Fig. 1) illustrate the consistent downregulation of Table 1 genes across each of the 5 data sets. Additional details on fold changes and p-values for the 24 genes appear in Supplementary Table 1 (lists of genes differentially expressed in 4 out of the 5 datasets are also provided, in Supplementary Table 8). While establishing such a stringent criterion may eliminate some relevant genes, we reasoned that the resulting genes would be unambiguously associated with AD. The intent of taking the intersection between multiple data sets, each imperfect and bearing its own peculiarities, is to compensate for shortcomings in any single data set. Indeed, the observed overlap in gene expression effects is striking, especially given that the analysis integrates data across multiple brain compartments and disease timepoints, as well as microarray platforms.

Pathway analysis was performed using DAVID^11^ on the list of 24 genes. These genes are enriched significantly in 19 GO CC pathways, including those related to mitochondria, membrane, and vesicles, specifically synaptic vesicle membrane, and 11 GO MF pathways, relating mainly to ATPase activity (Supplementary Table 6). Eight out of the 24 genes are annotated in GO CC as “mitochondrion” (GO:0005739): ATP5B, ATP6V1E1, BNIP3, C14orf2, GOT2, MRPS11, SLC25A11, and UQCRC1. Several additional genes are related to mitochondria, as will be discussed below. Four genes are annotated in GO MF as “ATPase activity, coupled to transmembrane movement of ions, phosphorylative mechanism” (GO:0015662): ATP5B, ATP6V1G2, ATP6V1E1, and ATP1A3. Three genes are annotated as “synaptic vesicle membrane” (GO:0030672): ATP6V1G2, SLC17A7, and SYP. Several genes on the list also have ties to glutamate (including CACNG3, which regulates AMPA-sensitive glutamate receptors; SLC17A7, which mediates glutamate uptake into synaptic vesicles; SLC25A11, a mitochondrial oxoglutarate carrier; and GOT2, mitochondrial glutamic-oxaloacetic transaminase 2), which is interesting because both impairments in glutamatergic transmission and excitotoxicity are thought to play a role in AD^12^.

### Degree of brain-specific expression of identified genes using BioGPS database

To determine the degree to which these genes were expressed preferentially in the brain, we utilized publicly available tissue-specific array data, as described in Methods. Overall, for the 24 genes identified, we found a high degree of specificity for expression in brain tissue (Fig. 1c).

### Searching the Gene Expression Omnibus detects gender differences in identified genes

To generate further insight into the list of expression-identified genes, we developed a method for identifying patterns in the Gene Expression Omnibus (GEO), a large database of publicly available gene expression data sets including over 500,000 human samples. We developed a Wilcoxon-test-based algorithm for comprehensively searching GEO to identify those samples in which each of the 24 genes was significantly modulated relative to other genes.

**Figure 2:**
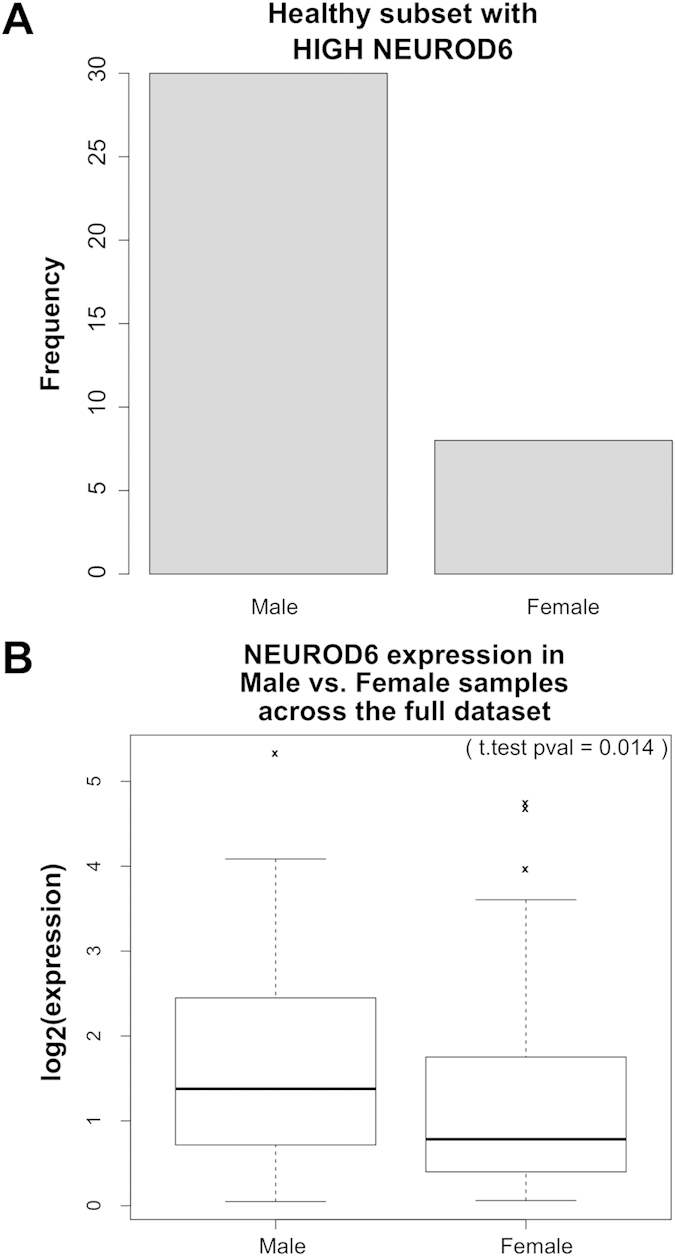
Possible link between NEUROD6 and gender. (**a**) Gender distribution of NEUROD6-high samples in a data set of healthy controls. (**b**) Comparison of NEUROD6 expression by gender across the entire data set of healthy controls.

The most striking finding was related to gender, a factor that is suggested to play a role in AD^13^. As shown in Supplementary Table 2, the CACNG3, GNG3, and NEUROD6 genes showed a gender-based pattern. For example, we observed in a dataset from healthy brain, GSE11882 (“Gene expression changes in the course of normal brain aging are sexually dimorphic”) that 38 samples were significantly enriched (FDR adjusted p-value < 0.05) for high expression of NEUROD6 (Supplementary Table 3). Strikingly, 30 of the 38 samples with enriched NEUROD6 expression were from males (Fig. 2a). This difference was highly significant, with hypergeometric p-value 1.71 × 10^−4^. In contrast, the entire GSE11882 data set was well-balanced by gender (173 samples with 82 female and 91 male) (Supplementary Fig. 2). We analyzed the pattern of NEUROD6 expression across the full data set to search for expression differences by gender in NEUROD6, and found that NEUROD6 expression was significantly higher in males than females with a nominal p-value of 0.014 (Fig. 2b). By dividing the data set into individual compartments, we found that NEUROD6 was significantly differentially expressed in two of the four compartments (with nominal p-values 0.0052 and 0.007; Supplementary Fig. 3). The finding that NEUROD6 differs in expression level between healthy men and women is particularly intriguing given that (a) NEUROD6 expression is downregulated with disease, and (b) gender may play a role in AD^13^.

Examining the GSE11882 data set with regard to the SNAP25 gene, two out of three expression probesets representing this gene were significantly lower in women than in men, in an individual compartment in the GSE11882 data set (nominal p-values 0.018 and 0.032; Supplementary Table 4). Note that GSE11882 was analyzed for differential gene expression between genders, but provided no Alzheimer-specific information.

To see whether these gender-related differences could have been recovered immediately from the original gene expression datasets, we also performed differential expression directly on each of these five AD datasets to examine differences in expression between genders (see Supplementary Table 9). We were not able to recover differences (beyond nominal p-values) between expression of NEUROD6 and SNAP25 in males and females in all five datasets (stratified by Alzheimer’s status, see Supplementary Table 11). In the largest dataset (GSE15222), we observed nominally significant differences in SNAP25 expression between females and males among AD patients but not among controls. In the second largest dataset (GSE44772), SNAP25 differed at least nominally by gender among AD patients and among controls. In one other dataset, GSE1297, SNAP25 differed at least nominally between M and F among controls. Fisher’s exact tests comparing the counts of samples originating from males and females, stratifying by disease status, determined that 4 out of the 5 datasets do not show a significant gender bias (see Supplementary Table 12). These results examining this more subtle effect are not surprising given the study sizes and different platforms across studies, and underscore the higher sensitivity of the pipeline described in this work as compared to traditional approaches for analysis.

We chose to focus on NEUROD6 and SNAP25 on the basis of these gender-related patterns and the genetic analyses described below.

### Utilizing SNP studies to identify putatively causative genes

To distinguish between downstream signals resulting from disease pathology, and upstream signals that may be more causative and therefore better targets for therapy, we utilized single-nucleotide polymorphism (SNP) data in conjunction with the gene expression data. The established method for combining these two types of data, expression quantitative trait locus (eQTL) analysis^14^, requires both gene expression and SNP data from the same cohort of patients. Since most of the available gene expression and SNP data came from separate cohorts, we developed another approach to identify converging lines of evidence for disease-causing genes from these disparate but complementary types of genomic data.

We examined the regions in and around (details as described below and in Methods) the 24 genes identified as downregulated in AD (Table 1) for disease-associated SNPs in three data sets: the ADNI1 cohort, the National Institute on Aging Late-Onset alzheimer’s Disease Family Study (“LOAD study”)^15^, and a recent study by Zhang *et al*. (“Cell study”)^3^. Limiting the SNPs of interest to targeted regions reduces the risk of false positives. This narrow focus supports the use of a more lenient p-value threshold greater than the typical threshold applied for GWAS studies (often p <  5 × 10^−8^). In order to relax the threshold from an overly conservative genomewide significance cutoff, yet maintain a desired level of stringency, we chose to use a p-value threshold of p < 5 × 10^−4^. The size of the region surrounding each gene was taken to be 200 kb up- and downstream, as many enhancer regions for transcriptional start sites, in general, are known to extend to such distances^16^. Indeed, in some cases, such elements are located as far as 1 Mb away from the affected gene; for example, in the case of the genes SHH^17^ and POU3F4^18^. For this reason, we performed an additional analysis using less stringent distance and p-value parameters of 1 Mb and p < 10^−2^, respectively. For both the strict and less stringent analyses, we defined a result to be of particular interest if a significant SNP from the GWAS studies comparing AD versus healthy control was located in the region of one of the AD-downregulated genes, in at least two out of three SNP studies.

In the case of the least stringent distance and p-value parameters (1 Mb and p < 10^−2^), for 15 out of 24 genes, significant SNPs were located within the region of interest in all three studies, as shown in Supplementary Table 7a, and the regions surrounding all but three of the expression-identified genes contained significant SNPs in at least two out of three studies. For further investigation, we utilized the results of the stringent filtering (p < 5 × 10^−4^, 200 kb; shown in Supplementary Table 7b) to help prioritize genes, as discussed in further detail below.

GWAS studies to date have identified APOE as the gene most consistently associated with AD. Carriers of the APOE ε4 (APOE4) allele have a significantly increased risk of AD^19^ for reasons that are incompletely understood, and several clinical studies have found putative differences in response to therapy based on APOE4 status^20^,^21^. In addition, recent work suggests that APOE4 imparts an increased risk of conversion to AD in women^22^. Given this information as well as the gender differences discussed above, in addition to examining the regions of interest in all patients, we also performed subset-specific analyses, based on both gender and apolipoprotein E (APOE) status. We reasoned that different subsets of AD patients might have differences in the biological factors driving their disease.

**Figure 3:**
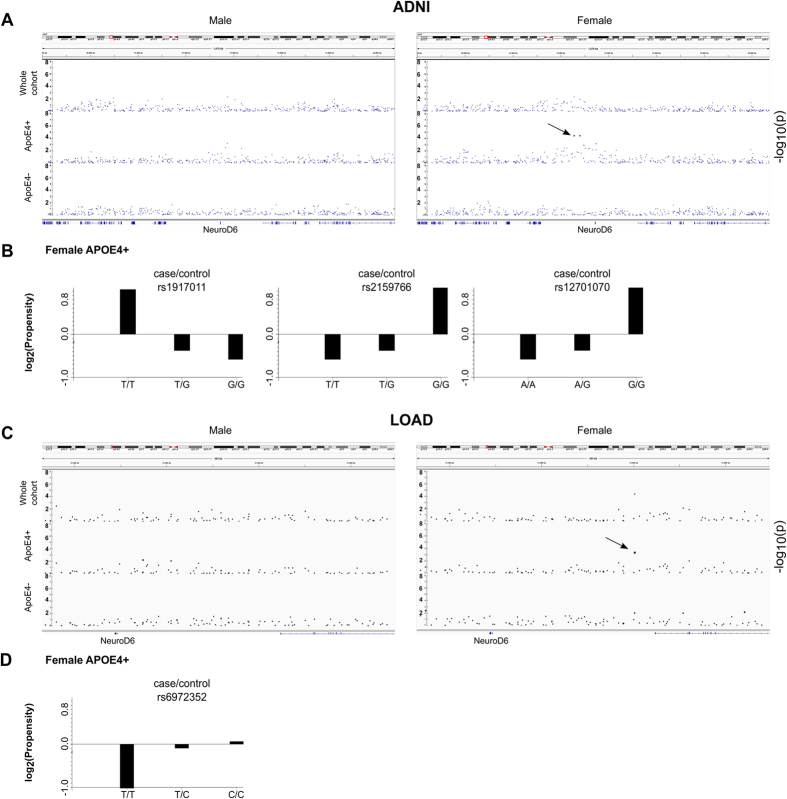
NEUROD6-related SNPs are associated with AD specifically in APOE4+ female patients. (**a**) Plot showing a “cone” of disease associated SNPs around NEUROD6 in APOE4+ female patients, but not in APOE4+ male, APOE4- female, or APOE4- male patients. Top AD-associated SNPs related to NEUROD6 in APOE4+ female patients in ADNI1 were: rs1917011 (p < 3.82e-5 in female APOE4+ patients, p < 0.692 in male APOE4+, p < 0.844 in female APOE4-), rs2159766 (p < 3.82e-5 in female APOE4+, p < 0.771 in male APOE4+, p < 0.624 in female APOE4-), and rs12701070 (p < 3.82e-5 in female APOE4+, p < 0.561 in male APOE4+, p < 0.624 in female APOE4-). (**b**) Propensity plots showing the disease risk (positive values) or protection (negative values) in APOE4+ female patients as a function of a patient’s status for each of three top NEUROD6 SNPs. (**c**) Plot showing disease associated SNP near NEUROD6 in APOE4+ female patients, but not in APOE4+ male, APOE4- female, or APOE4- male patients from the LOAD data set. This SNP was rs6972352 (p < 0.00049 in female APOE4+, p < 0.2247 in male APOE4+, and p < 0.010 in female APOE4-). (**d**) Propensity plots showing the disease risk (positive values) or protection (negative values) in APOE4+ female patients as a function of a patient’s status for each of three top NEUROD6 SNPs.

SNPs in the region of NEUROD6 were associated with AD specifically in APOE4+ women in both the ADNI1 and LOAD cohorts (Supplementary Table 7b). These NEUROD6 SNPs are illustrated in Fig. 3a,c. We utilized a newly developed propensity plotting method to visualize the specific influence of these SNPs (Fig. 3b,d). Status at these SNPs was highly associated with disease propensity. In contrast, SNPs in the region of SNAP25 were associated with AD specifically in APOE4+ men in both the LOAD and Cell data sets (Supplementary Table 7b and Supplementary Fig. 4). SNPs were also identified near MRPS11 for APOE4+ women, and near GOT2 for women as well as for the whole cohort, for the chosen distance and p-value parameters. We narrowed our focus to NEUROD6 and SNAP25 since their respective identified SNPs were closer to these genes than to MRPS11 and GOT2, though all of these genes may have intriguing biological connections, as discussed below.

### Identifying medicines to restore expression of causative genes, using CMAP data

We next sought to determine which medicinal drugs and compounds could restore expression of NEUROD6 or SNAP25 shown to be downregulated in AD. We developed an enrichment algorithm to identify specific drugs in the Connectivity Map (CMAP)^23^ databases that induced significantly higher expression of NEUROD6 or SNAP25 in culture. We identified 34 unique compounds that upregulate NEUROD6, and nine that upregulate SNAP25 (Table 2). Note that this hypothesis-generating analysis serves to identify drugs of interest for further examination, and these compounds are not expected to be highly specific for these genes. The full list of drugs with their known gene/pathway targets is available in Supplementary Table 10.

## Discussion

A number of the genes identified in the expression analysis as downregulated with AD have striking common biology. The list of 24 genes enriched significantly for GO pathways including those related to mitochondria, membrane, and vesicles, specifically synaptic vesicle membrane, and ATPase activity (Supplementary Table 6). Several genes on the list also have ties to glutamate, which is of particular interest since both impairments in glutamatergic transmission and excitotoxicity are thought to play a role in AD^12^. To evaluate which genes on this list were likely to be causal in disease, we examined SNP data and determined that in particular patient cohorts, significant SNPs were found in multiple data sets in the regions around NEUROD6 and SNAP25.

NEUROD6 is a transcription factor involved in neuronal differentiation, and has been shown to increase mitochondrial mass and play a role in response to oxidative stress^24^. This is intriguing because the aging process has a negative impact on mitochondrial function^25^ and leads to an increase in mitochondrial DNA mutations^26^, and rates of Alzheimer’s increase dramatically with age^27^.

APOE also has ties to the mitochondria^28^. The APOE ε4 isoform has been shown to cause mitochondrial damage specifically in neurons^28^. APOE ε4 also has lower antioxidant capability than other isoforms, and amyloid beta induces oxidative stress to a greater extent when APOE ε4 is present^29^. Oxidative stress may also induce hyperphosphorylation of tau^30^, another key factor in AD. Impairing the transport of mitochondria into axons was shown to enhance tau phosphorylation and neurodegeneration^31^. Several studies in a variety of systems have shown that oxidative stress induces upregulation of BACE1^32^, an enzyme critical for the production of amyloid beta. In other systems, oxidative stress has been shown to increase production of amyloid precursor protein^33^. Because NEUROD6 confers tolerance to oxidative stress^24^, it has the potential to mitigate some of this damage. As NEUROD6 expression is lower in women, and APOE4+ individuals have lowered tolerance for ROS damage^28^,^29^, it stands to reason that a SNP associated with further impairment of NEUROD6 may put APOE4+ females at particular risk of damage due to oxidative stress.

SNAP25 has an important role in synaptic function as part of the Soluble NSF Attachment Protein Receptor (SNARE) complex, which is involved in synaptic vesicle exocytosis, and has been tied to neurodegeneration^34^,^35^. SNARE proteins are known to be sensitive to oxidative stress, with SNAP25 being the most sensitive, which has been proposed to relate mitochondrial dysfunction to reduced synaptic activity in neurodegeneration^36^. Indeed, in a mouse model of AD using male mice, lower SNAP25 levels were observed in the hippocampus, along with lowered levels of glutamate, ATP, and mitochondrial membrane potential; the authors attributed the observed deficiency in glutamatergic neurotransmission (which requires a high level of energy consumption) to dysfunction of mitochondrial bioenergetics^37^.

The other two genes identified for the chosen distance and p-value parameters for certain patient subsets (female APOE4+, and female as well as whole cohort, respectively) were MRPS11 and GOT2. Intriguingly, both are mitochondrial genes. MRPS11 encodes a component of the small subunit of the mitochondrial ribosome. Mutations in other mitochondrial small subunit ribosomal proteins are known to cause severe disruption in the respiratory chain^38^. GOT2 encodes a mitochondrial kynurenine transaminase, which acts on kyurenine to produce kynurenic acid (KYNA), a neuroprotective NMDA and nicotinic receptor antagonist that may also have antioxidant properties^39^ and can affect glutamatergic neurotransmission in multiple ways^12^. Interestingly, lower KYNA levels have been observed in AD patients^40^, and raising KYNA levels via inhibition of kyurinine-3-monooxygenase (KMO; another enzyme that acts on kyurenine) ameliorates neurodegeneration *in vivo*^41^.

Several of the compounds identified via our CMAP search as upregulating NEUROD6 or SNAP25 expression have shown promise in published mouse models of AD, as described below. The highest ranked compound for its ability to upregulate NEUROD6 is sodium phenylbutyrate, which has recently been proposed as a therapeutic for neurodegenerative diseases due to its ability to increase neurotrophic factors in brain cells, along with the fact that it is safe, orally delivered, and crosses the blood brain barrier^42^. Another study using a mouse model of AD demonstrated that independent of Aβ levels, sodium phenylbutyrate decreased spatial learning and memory impairment by upregulating markers for synapatic and dendritic growth^43^. The third most highly ranked compound is 2-Deoxy-D-Glucose, which has been shown to reduce pathology in a female mouse model of AD by inducing ketogenesis and enhancing mitochondrial capacity^44^. We propose that these drugs warrant additional investigation as AD therapeutics, particularly in APOE4+ women.

Why is NEUROD6 expression most significant in females? Estrogen signaling appears to stimulate the production of enzymes such as glutathione peroxidase that protect the mitochondria against oxidative stress^13^, so the loss of estrogen upon age could leave women more susceptible to mitochondrial damage associated with impairment of NEUROD6 production and the resultant loss of protective effects. In fact, among the list of compounds from CMAP2 that most significantly elevate expression of NEUROD6 (Table 2) is genistein, which has been proposed as a means to replace the protective effect of estrogen on mitochondria in aging women^13^.

The top drug shown to significantly elevate SNAP25 expression, valproic acid, is known to have neuroprotective properties^45^. In recent studies in mouse models of AD, valproic acid was demonstrated to protect against loss of neurons^46^ and limit Aβ production and behavioral deficits^47^. Karakoline is a nicotinic receptor agonist that was recently shown to improve cognitive function in a mouse model of AD^48^. Tetracycline was shown to protect from Aβ toxicity in C elegans^49^, and its derivatives are actively being explored as potential therapeutics in mouse models of AD^50^. We hypothesize that these compounds warrant further investigation for treatment of AD, particularly in APOE4+ men.

**Figure 4:**
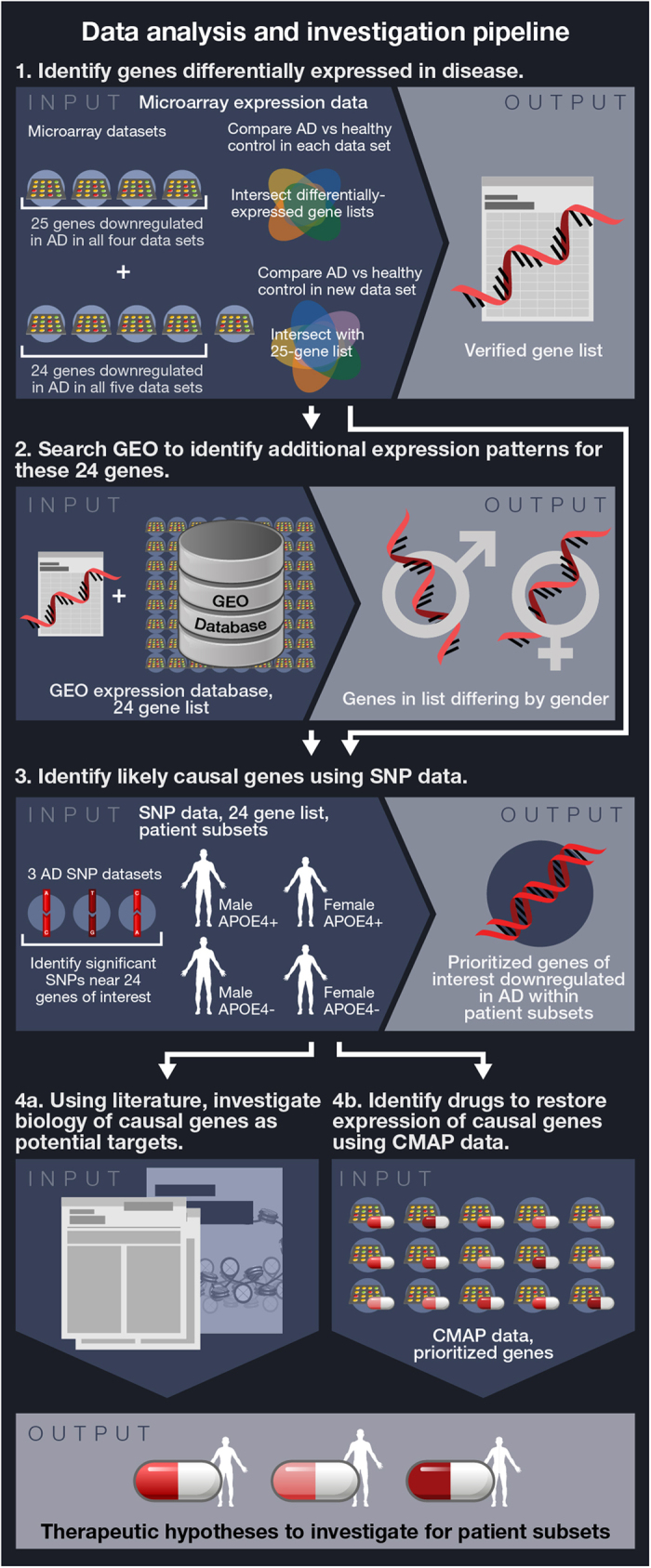
Schematic of analysis pipeline. (Art credit: Kate Mahan.) We developed an innovative pipeline for extracting new insights from existing publicly available resources, including five gene expression datasets, three SNP datasets, and multiple databases, in order to generate hypotheses for potential treatment strategies in subsets of AD patients. (**1**) We identified genes differentially expressed in AD in multiple gene expression data sets, leading to a list of 24 genes downregulated in AD. (**2**) We searched all of GEO to identify expression patterns for these genes in additional (not necessarily AD) datasets, revealing gender differences for some genes. (**3**) For patient subsets of interest (defined by gender and APOE4 status), we performed GWAS in each of three separate SNP data sets (AD vs healthy control) in regions up and downstream of 24 genes, allowing prioritization of genes having nearby SNPs. (**4**) The genes thus identified provide insights that can help facilitate development of new therapeutics. For example, searching the CMAP database identified existing drugs warranting further investigation for specific subsets of AD patients. In addition, further elucidating the biology of these genes can allow identification of entirely new therapeutic targets.

We developed an innovative pipeline for extracting new insights from existing data sets, utilizing a breadth of separate, publicly available resources (as illustrated in Fig. 4). This pipeline is innovative in particular because of the focus on integrating multiple gene expression datasets across various platforms in order to achieve a robust intersection of disease-affected genes, then leveraging these results in combination with genetic studies in order to identify those that are more likely causal. This approach has identified NEUROD6 and SNAP25 as important factors in AD, especially in APOE4+ women and men, respectively. Both of the genes identified have solid connections to the neuronal oxidative damage response. We have also identified subset-specific drug candidates for modulation of these genes. More broadly, our analyses suggest that further exploration of gender-specific biology could lead to effective new medicines for neurodegeneration, along with special considerations for corresponding clinical trials, and that patient stratification by gender, genetics or other factors could facilitate data-driven identification of new therapeutic options for a variety of diseases.

## Methods

### Identification, Processing and Analysis of Expression Data Sets

All expression data sets used in this analysis were obtained from the Gene Expression Omnibus (GEO) site (http://www.ncbi.nlm.nih.gov/geo/) and consisted of GEO project accession numbers: GSE5281^51^, GSE1297^52^, GSE36980^53^, GSE15222^54^, and GSE44772^3^. A summary of these data sets, including all sample IDs used in the analysis, array type, and the compartment of the brain that the samples were collected from can be found in Supplementary Table 5. Each data set was processed and analyzed independently. We identified genes differentially expressed between AD and control in each using LIMMA (Version 3.18.13)^55^, taking a significance threshold of FDR-corrected p-value < 0.05. We further identified the genes significant in multiple data sets by taking the intersection of the individual differentially expressed gene lists across studies.

The general analysis method employed for expression data was as follows. CEL files were obtained from GEO (except where noted) and the data were RMA normalized^56^. The probesets determined to be significantly different between the two conditions of interest (in nearly all cases, AD vs control, except for GSE1297 where this condition was based on low versus high NFT score, as noted below) were calculated using LIMMA. For chips with available MM/PM (mismatch/perfect match) probe data (Affy U133A or U133 Plus 2.0 chips), results were filtered according to Presence/Absence (P/A) calls computed in R using the “affy” package^57^, such that the average P/A call for all of the samples in the condition of interest with the higher average value (e.g., controls, for probesets downregulated in disease) was at least marginal.

LIMMA comparisons were used as a metric for assessing the signal to noise tradeoff in each brain compartment for data sets with multiple brain compartments (visual cortex excluded). The compartment with the greatest number of significant probesets (FDR p-value < 0.05) was chosen for analysis. For Affymetrix chips, the presence of Affymetrix control probesets among the top significant differential expression results was considered a QC flag; compartments having Affymetrix control probesets as either >0.25% of significant differential expression results, or in any of the top ten results, were discarded.

GSE5281 consists of samples collected from 6 compartments of the brains of AD patients along with controls and measured using the Affymetrix Human Genome U133 Plus 2.0 Array. We used the samples collected from the Entorhinal Cortex (EC), per the QC procedure described above.

GSE1297 contains mini-mental state examination (MMSE) scores and neurofibrillary tangle (NFT) scores for all individuals in the study, with gene expression measurements from the hippocampus made using the Affymetrix Human Genome U133A Array. We found roughly 5 times as many significant probesets when comparing the individuals with the 9 highest NFT scores versus the individuals with the 9 lowest NFT scores than attained when comparing Severe AD patients (based on MMSE score) vs. controls. For this reason, we used samples with the top 9 NFT scores versus the lowest 9 NFT scores in this analysis. The lowest 9 NFT scores included both controls and patients labeled as having incipient AD. The highest 9 NFT scores included patients labeled as having severe AD, moderate AD, and incipient AD.

GSE36980 is comprised of samples from 3 compartments of the brains of AD patients and controls, measured using the Affymetrix Human Gene 1.0 ST Array. We used the data from the hippocampus as the compartment with the most probesets significantly differing in expression between AD patients and controls (as described above).

GSE15222 consists of samples collected from multiple cortical compartments of the brains of AD patients and controls, measured using the Sentrix HumanRef-8 Expression BeadChip. Raw data was not available from this data set, so we used the processed data available on the corresponding author’s laboratory website^58^. The authors rank-invariant normalized their data and then filtered it in two ways. First, transcripts were only considered if they were detected in at least 90% of AD patients or 90% of controls. Second, transcript expression intensities were only considered in analyses for a given sample if their Illumina detections scores were >0.99^58^. Since these data had been normalized across all measured compartments of the brain, we considered all the samples together.

The net result of these analyses was a list of probesets significantly affected by Alzheimer’s disease for each data set. To obtain a robust list of affected genes, we determined the intersection of the gene names from all four lists. We performed separate intersections for genes upregulated in each study and genes downregulated in each study. After obtaining a list of 25 genes downregulated in disease relative to control across all four studies, we obtained a fifth data set (GSE44772).

GSE44772, which we obtained after analyzing the other four data sets, consists of samples collected from three compartments in the brains of both AD patients and controls, measured using a custom Rosetta/Merck Human 44k 1.1 microarray. Using the same QC procedure described above, we chose to utilize the data from the prefrontal cortex. Raw data was not available from this data set, so we used the processed data available in the Series Matrix File on GEO. The data was in the form of normalized log10 ratios between the test sample and a pooled reference sample. LIMMA was used to determine the probesets that were significantly different between the AD patients and the controls. 24 out of the 25 downregulated genes from the four-data set intersection were also significantly downregulated with disease in this data set.

### Brain specificity heat maps

For the 24 genes of interest, all probesets were mapped to tissue-specific arrays^59^, available via BioGPS^60^. The probesets were clustered hierarchically with a metric of Pearson correlation, and displayed via subtracting by the median and dividing by the absolute deviation (both in GENE-E, http://www.broadinstitute.org/cancer/software/GENE-E/). Tissues were annotated by whether or not they were brain-associated, and sorted and grouped accordingly.

### Stratified GWAS

We conducted targeted gene association testing from the SNP data sets listed in the text using PLINK^61^, with patient subsets defined by gender and APOE4 status. Results were visualized using the Integrative Genomics Viewer (IGV)^62^.

In order to obtain the strongest possible AD-relevant signals, we restricted the analysis of the LOAD data set to only those patients with an AD diagnosis confirmed by autopsy. Similarly, in analysis of the Zhang *et al*. data set we excluded controls and patients having a diagnosis of Huntington’s disease.

Of the 757 patients in ADNI1 who were genotyped via Illumina 610 Quad array SNP chip, 389 were categorized as either AD patients or healthy controls, and the rest (those with mild cognitive impairment, MCI) were excluded from further consideration. GWAS was also run on patient sub-cohorts, stratified by APOE4 status, gender, and the combination of both. Effect sizes for each group are as follows: unstratified = 175 AD, 214 healthy controls (HC); APOE4- = 58 AD, 156 HC; APOE4+ = 117 AD, 58 HC; Female APOE4- = 31 AD, 73 HC; Female APOE4+ = 51 AD, 26 HC; Male APOE4- = 27 AD, 88 HC; Male APOE4+ = 66 AD, 32 HC; Male all = 93 AD, 115 HC; Female all = 82 AD, 99 HC.

The LOAD data set included 1985 patients and 2058 controls genotyped via Illumina Human 610 Quad v1B SNP chip. Patients included in the analysis were limited to those who had an AD diagnosis confirmed by autopsy. Unstratified, subjects included in the analysis were 440 patients and 2058 controls. Numbers of subjects within subsets were: APOE4- = 99 AD, 1256 HC; APOE4+ = 341 AD, 802 HC; Female APOE4- = 74 AD, 773 HC; Female APOE4+ = 230 AD, 483 HC; Male APOE4- = 25 AD, 483 HC; Male APOE4+ = 111 AD, 319 HC; Male all = 136 AD, 802 HC; Female all = 304 AD, 1256 HC.

The Zhang *et al*. (or “CELL”) data set included 374 patients and 366 controls genotyped via Illumina HumanHap650Y SNP chip. We excluded subjects with a diagnosis of Huntington’s disease from the analysis. Unstratified, 371 patients and 159 controls were included in the analysis. Numbers of subjects in subsets were as follows: APOE4- = 209 AD, 130 HC; APOE4+ = 162 AD, 29 HC; Female APOE4- = 129 AD, 34 HC; Female APOE4+ = 90 AD, 5 HC; Male APOE4- = 80 AD, 96 HC; Male APOE4+ = 72 AD, 24 HC; Male all = 152 AD, 120 HC; Female all = 219 AD, 39 HC. In this data set we removed from consideration patients and controls with a diagnosis of Huntington’s disease. Therefore, smaller numbers of controls were available, especially in the highly stratified subsets. Specifically, the female APOE+ cohort contained only five controls, which may explain why SNPs calculated to be significant in female APOE4+ patients in the other two data sets did not achieve significance in this subset.

### Propensity plotting

We adapt a measure of bias for identifying amino acids on the surface that are likely to be part of an interface domain from the proteomics field^63^,^64^,^65^ to measure the preference of a particular SNP genotype to case vs. control subsets of the data set. Briefly, we define SNP propensity to be the fraction of patients diagnosed with AD given a particular SNP variant to the control fraction with this SNP variant. Specifically, it is defined as:
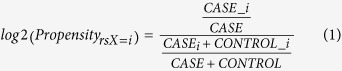
Where *CASE*_*i*_ is the fraction of patients diagnosed with AD and having SNP variant *i*. *CONTROL*_*i*_ is the fraction of control individuals with SNP variant *i*. *CASE* is the total number of patients diagnosed with AD in the data set, and CONTROL is the total number of control individuals in the data set.

### GEO-search analysis

We searched all human data sets across all platforms in GEO as of July 2013. We used a one-sample Wilcoxon test to measure the significance of differential expression for probesets annotated to a gene of interest against all other probesets in the sample, correcting for FDR using Benjamini-Hochberg.

Supplementary Table 2 was constructed by counting the number of samples, m, from the GSE11882 data set that passed our adjusted p-value threshold of 0.05 for the GEO search algorithm described above. Of the samples that passed our adjusted threshold of 0.05, we counted the number of samples, n, that came from males. We then calculated the significance of obtaining ≥ n male samples if we drew m samples randomly from a total of 173 samples (which is the total number of samples in GSE11882) with 91 males (total number of male samples in GSE11882) based on the hypergeometric distribution. We omitted the significance calculation if the number of significant samples, m, was less than 15 (<10% of the data set).

### CMAP search

CMAP (Connectivity Map) is a large collection of microarray-based transcriptional signatures for 7000 expression profiles from cultured cells treated with 1,309 compounds. We obtained the full CMAP (builds 01 and 02)^66^,^23^ data sets and utilized a one-sample Wilcoxon test to identify expression profiles from compounds that significantly increased expression of a gene of interest in culture after treatment. We then adjusted for multiple hypothesis testing (FDR p-value < 0.05).

### ADNI-data acquisition

The SNP data used in the preparation of this article were obtained from the Alzheimer’s Disease Neuroimaging Initiative (ADNI) database (adni.loni.ucla.edu). The ADNI was launched in 2003 by the National Institute on Aging (NIA), the National Institute of Biomedical Imaging and Bioengineering (NIBIB), the Food and Drug Administration (FDA), private pharmaceutical companies and non-profit organizations, as a $60 million, 5-year public- private partnership. The primary goal of ADNI has been to test whether serial magnetic resonance imaging (MRI), positron emission tomography (PET), other biological markers, and clinical and neuropsychological assessment can be combined to measure the progression of mild cognitive impairment (MCI) and early Alzheimer’s disease (AD). Determination of sensitive and specific markers of very early AD progression is intended to aid researchers and clinicians to develop new treatments and monitor their effectiveness, as well as lessen the time and cost of clinical trials. The Principal Investigator of this initiative is Michael W. Weiner, MD, VA Medical Center and University of California – San Francisco. ADNI is the result of efforts of many co- investigators from a broad range of academic institutions and private corporations, and subjects have been recruited from over 50 sites across the U.S. and Canada. The initial goal of ADNI was to recruit 800 subjects but ADNI has been followed by ADNI-GO and ADNI-2. To date these three protocols have recruited over 1500 adults, ages 55 to 90, to participate in the research, consisting of cognitively normal older individuals, people with early or late MCI, and people with early AD. The follow up duration of each group is specified in the protocols for ADNI-1, ADNI-2 and ADNI-GO. Subjects originally recruited for ADNI-1 and ADNI-GO had the option to be followed in ADNI-2. For up-to-date information, see www.adni-info.org.

## Additional Information

**How to cite this article**: Fowler, K. D. *et al*. Leveraging existing data sets to generate new insights into Alzheimer's disease biology in specific patient subsets. *Sci. Rep*. **5**, 14324; doi: 10.1038/srep14324 (2015).

## Supplementary Material

Supplementary Information

## Figures and Tables

**Table 1:** List of 24 genes downregulated in AD consistently across 5 data sets.

Downregulated in AD vs control in all 5 data sets			
AP3B2	C14orf132	MRPS11	SLC25A11
ATP1A3	C14orf2	NEUROD6	SNAP25
ATP5B	CACNG3	PPP1R11	SYP
ATP6V1E1	GNG3	PTPRN2	TPI1
ATP6V1G2	GOT2	RGS7	UQCRC1
BNIP3	MAGED1	SLC17A7	YWHAB

**Table 2:** Drug candidates determined based on patient subset analysis.

CMAP compound name	p.val	adj.p.val
**(A) NEUROD6-elevating compounds**		
sodium phenylbutyrate	8.03E-05	4.23E-02
arachidonic acid	8.22E-05	4.23E-02
2-deoxy-D-glucose	8.59E-05	4.23E-02
fasudil	8.76E-05	4.23E-02
nordihydroguaiaretic acid	1.04E-04	4.23E-02
monastrol	1.09E-04	4.23E-02
tacrolimus	1.12E-04	4.23E-02
quercetin	1.12E-04	4.23E-02
sulindac	1.14E-04	4.23E-02
troglitazone	1.17E-04	4.23E-02
staurosporine	1.17E-04	4.23E-02
troglitazone	1.22E-04	4.23E-02
thalidomide	1.26E-04	4.23E-02
CP-944629	1.35E-04	4.23E-02
mercaptopurine	1.40E-04	4.23E-02
haloperidol	1.49E-04	4.23E-02
exisulind	1.57E-04	4.23E-02
sirolimus	1.71E-04	4.23E-02
tanespimycin	1.71E-04	4.23E-02
suramin sodium	1.74E-04	4.23E-02
genistein	1.76E-04	4.23E-02
erastin	1.78E-04	4.23E-02
clofibrate	1.80E-04	4.23E-02
LY-294002	1.92E-04	4.23E-02
tanespimycin	1.93E-04	4.23E-02
LY-294002	1.97E-04	4.23E-02
prednisolone	1.99E-04	4.23E-02
fulvestrant	2.01E-04	4.23E-02
meteneprost	2.05E-04	4.23E-02
monorden	2.17E-04	4.23E-02
tretinoin	2.22E-04	4.23E-02
nifedipine	2.30E-04	4.23E-02
sulindac sulfide	2.32E-04	4.23E-02
wortmannin	2.36E-04	4.23E-02
MK-886	2.46E-04	4.29E-02
PF-01378883-00	2.59E-04	4.38E-02
monorden	2.82E-04	4.65E-02
iloprost	3.06E-04	4.91E-02
*Note: 34 unique compounds. Repeats due to differing cell lines or repeated experiments*.		
**(B) SNAP25-elevating compounds**		
valproic acid	2.20E-05	1.91E-02
guanabenz	9.14E-05	3.81E-02
karakoline	8.89E-05	3.81E-02
tetracycline	1.03E-04	4.01E-02
diloxanide	1.28E-04	4.45E-02
metoprolol	1.38E-04	4.52E-02
yohimbic acid	1.59E-04	4.75E-02
azapropazone	1.63E-04	4.75E-02
proguanil	1.93E-04	4.92E-02

(a) Compounds that induce enriched expression of NEUROD6. (b) Compounds that induce enriched expression of SNAP25.
