# Supplementary material for: Leveraging existing data sets to generate new insights into Alzheimer’s disease biology in specific patient subsets
Source: Sci Rep. 2015 Sep 23;5:14324. doi: 10.1038/srep14324 (PMC4585817; doi:10.1038/srep14324)
Supplement: Supplementary Information [file srep14324-s1.pdf]

Supplementary Material for:

Leveraging existing data sets to generate new insights into Alzheimer's disease biology in specific patient subsets

Authors

Kevin D. Fowler,[1*] Jason M. Funt,[1*] Maxim N. Artyomov,[1,2] Benjamin Zeskind,[1] Sarah E. Kolitz,[1+] Fadi Towfic,[1+] for the Alzheimer's Disease Neuroimaging Initiative[3]

Affiliations

[1]Immuneering Corporation, Cambridge, Massachusetts, United States of America

[2]Department of Immunology and Pathology, Washington University in St. Louis, St. Louis, Missouri, United States of America

[3]SNP data used in preparation of this article were obtained from the Alzheimer's Disease Neuroimaging Initiative (ADNI) database (adni.loni.ucla.edu). As such, the investigators within the ADNI contributed to the design and implementation of ADNI and/or provided data but did not participate in analysis or writing of this report. A complete listing of ADNI investigators can be found below, and at: http://adni.loni.ucla.edu/wp-content/uploads/how_to_apply/ADNI_Acknowledgement_List.pdf.

[*,+] These authors contributed equally.

Correspondence should be addressed to B.Z. (bzeskind@immuneering.com, Tel: +1 617-500-8080 ext. 121, Fax: +1 617-449-9588)

Alzheimer's Disease Neuroimaging Initiative

ADNI Investigators:

Michael W. Weiner (1), Paul Aisen (2), Ronald Petersen (3), Clifford R. Jack Jr. (3), William Jagust (4), John Q. Trojanowki (5), Arthur W. Toga (6), Laurel Beckett (7), Robert C. Green (8), Andrew J. Saykin (9), John Morris (10), Leslie M. Shaw (5), Zaven Khachaturian (11), Greg Sorensen (12), Lew Kuller (13), Marc Raichle (10), Steven Paul (14), Peter Davies (15), Howard Fillit (16), Franz Hefti (17), Davie Holtzman (10), M. Marcel Mesulam (18), William Potter (19), Peter Snyder (20), Adam Schwartz (21), Tom Montine (22), Ronald G. Thomas (2), Michael Donohue (2), Sarah Walter (2), Devon Gessert (2), Tamie Sather (2), Gus Jiminez (2), Archana B. Balasubramanian (2), Jennifer Mason (2), Iris Sim (2), Danielle Harvey (7), Matthew Bernstein (3), Nick Fox (23), Paul Thompson (24), Norbert Schuff (1), Charles DeCarli (7), Bret Borowski (25), Jeff Gunter (25), Matt Senjem (25), Prashanthi Vemuri (25), David Jones (25), Kejal Kantarci (25), Chad Ward (25), Robert A. Koeppe (26), Norm Foster (27), Eric M. Reiman (28), Kewei Chen (28), Chet Mathis (29), Susan Landau (4), Nigel J. Cairns (10), Erin Householder (10), Lisa Taylor-Reinwald (10), Virginia Lee (30), Magdalena Korecka (30), Michal Figurski (30), Karen Crawford (6), Scott Neu (6), Tatiana M. Foroud (9), Steven Potkin (31), Li Shen (9), Kelley Faber (9), Sungeun Kim (9), Kwangsik Nho (9), Leon Thal (2), Neil Buckholtz (32), Marilyn Albert (33), Richard Frank (34), John Hsiao (32), Jeffrey Kaye (35), Joseph Quinn (35), Lisa Silbert (35), Betty Lind (35), Raina Carter (35), Sara Dolen (35), Lon S. Schneider (6), Sonia Pawluczyk (6), Mauricio Beccera (6), Liberty Teodoro (6), Bryan M. Spann (6), James Brewer (2), Helen Vanderswag (2), Adam Fleisher (2), Judith L. Heidebrink (26), Joanne L. Lord (26), Sara S. Mason (3), Colleen S. Albers (3), David Knopman (3), Kris Johnson (3), Rachelle S. Doody (36), Javier Villanueva-Meyer (36), Munir Chowdhury (36), Susan Rountree (36), Mimi Dang (36), Yaakov Stern (37), Lawrence S. Honig (37), Karen L. Bell (37), Beau Ances (10), Maria Carroll (10), Mary L. Creech (10), Erin Franklin (10), Mark A. Mintun (10), Stacy Schneider (10), Angela Oliver (10), Daniel Marson (38), Randall Griffith (38), David Clark (38), David Geldmacher (38), John Brockington (38), Erik Roberson (38), Marissa Natelson Love (38), Hillel Grossman (39), Effie Mitsis (39), Raj C. Shah (40), Leyla deToledo-Morrell (40), Ranjan Duara (41), Daniel Varon (41), Maria T. Greig (41), Peggy Roberts (41), Chiadi Onyike (33), Daniel D'Agostino II (33), Stephanie Kielb (33), James E. Galvin (42), Brittany Cerbone (42), Christina A. Michel (42), Dana M. Pogorelec (42), Henry Rusinek (42), Mony J de Leon (42), Lidia Glodzik (42), Susan De Santi (42), P. Murali Doraiswamy (43), Jeffrey R. Petrella (43), Terence Z. Wong (43), Edward Coleman (43), Steven E. Arnold (5), Jason H. Karlawish (5), David Wolk (5), Christopher M. Clark (5), Charles D. Smith (44), Greg Jicha (44), Peter Hardy (44), Partha Sinha (44), Elizabeth Oates (44), Gary Conrad (44), Oscar L. Lopez (29), MaryAnn Oakley (29), Donna M. Simpson (29), Anton P. Porsteinsson (45), Bonnie S. Goldstein (45), Kim Martin (45), Kelly M. Makino (45), M. Saleem Ismail (45), Connie Brand (45), Ruth A. Mulnard (31), Gaby Thai (31), Catherine Mc-Adams-Ortiz (31), Kyle Womack (46), Dana Mathews (46), Mary Quiceno (46), Allan I. Levey (47), James J. Lah (47), Janet S. Cellar (47), Jeffrey M. Burns (48), Russell H. Swerdlow (48), William M. Brooks (48), Liana Apostolova (49), Kathleen Tingus (49), Ellen Woo (49),

Daniel H.S. Silverman (49), Po H. Lu (49), George Bartzokis (49), Neill R Graff-Radford (50), Francine Parfitt (50), Tracy Kendall (50), Heather Johnson (50), Martin R. Farlow (9), Ann Marie Hake (9), Brandy R. Matthews (9), Jared R. Brosch (9), Scott Herring (9), Cynthia Hunt (9), Christopher H. van Dyck (51), Richard E. Carson (51), Martha G. MacAvoy (51), Pradeep Varma (51), Howard Chertkow (52), Howard Bergman (52), Chris Hosein (52), Sandra Black (53), Bojana Stefanovic (53), Curtis Caldwell (53), Ging-Yuek Robin Hsiung (54), Howard Feldman (54), Benita Mudge (54), Michele Assaly (54), Elizabeth Finger (55), Stephen Pasternack (55), Irina Rachinsky (55), Dick Drost (55), Andrew Kertesz (55), Charles Bernick (56), Donna Munic (56), Marek-Marsel Mesulam (18), Kristine Lipowski (18), Diana Kerwin (18), Chuang-Kuo Wu (18), Nancy Johnson (18), Carl Sadowsky (57), Teresa Villena (57), Raymond Scott Turner (58), Kathleen Johnson (58), Brigid Reynolds (58), Reisa A. Sperling (59), Keith A. Johnson (59), Gad Marshall (59), Jerome A. Yesavage (60), Joy L. Taylor (60), Barton Lane (60), Allyson Rosen (60), Jared Tinklenberg (60), Marwan N. Sabbagh (61), Christine M. Belden (61), Sandra A. Jacobson (61), Sherye A. Sirrel (61), Neil Kowall (62), Ronald Killiany (62), Andrew E. Budson (62), Alexander Norbash (62), Patricia Lynn Johnson (62), Thomas O. Obisesan (63), Saba Wolday (63), Joanne Allard (63), Alan Lerner (64), Paula Ogrocki (64), Curtis Tatsuoka (64), Parianne Fatica (64), Evan Fletcher (65), Pauline Maillard (65), John Olichney (65), Owen Carmichael (65), Smita Kittur (66), Michael Borrie (67), T-Y Lee (67), Rob Bartha (67), Sterling Johnson (68), Sanjay Asthana (68), Cynthia M. Carlsson (68), Steven G. Potkin (31), Adrian Preda (31), Dana Nguyen (31), Pierre Tariot (28), Anna Burke (28), Nadira Trncic (28), Adam Fleisher (28), Stephanie Reeder (28), Vernice Bates (69), Horacio Capote (69), Michelle Rainka (69), Douglas W. Scharre (70), Maria Kataki (70), Anahita Adeli (70), Earl A. Zimmerman (71), Dzintra Celmins (71), Alice D. Brown (71), Godfrey D. Pearlson (72), Karen Blank (72), Karen Anderson (72), Laura A. Flashman (73), Marc Seltzer (73), Mary L. Hynes (73), Robert B. Santulli (73), Kaycee M. Sink (74), Leslie Gordineer (74), Jeff D. Williamson (74), Pradeep Garg (74), Franklin Watkins (74), Brian R. Ott (75), Henry Querfurth (75), Geoffrey Tremont (75), Stephen Salloway (76), Paul Malloy (76), Stephen Correia (76), Howard J. Rosen (1), Bruce L. Miller (1), David Perry (1), Jacobo Mintzer (77), Kenneth Spicer (77), David Bachman (77), John Rogers (78), Nunzio Pomara (79), Raymundo Hernando (79), Antero Sarrael (79), Susan K. Schultz (80), Laura L. Boles Ponto (80), Hyungsub Shim (80), Karen Elizabeth Smith (80), Norman Relkin (14), Gloria Chaing (14), Michael Lin (14), Lisa Ravdin (14), Amanda Smith (81), Balebail Ashok Raj (81), Kristin Fargher (81), Thomas Neylan (1), Jordan Grafman (82), Melissa Davis (2), Rosemary Morrison (2), Jacqueline Hayes (1), Shannon Finley (1), Karl Friedl (83), Debra Fleischman (40), Konstantinos Arfanakis (40), Olga James (43), Dino Massoglia (77), Olga Brawman-Mintzer (77), Walter Martinez (57), Kelly Behan (58), J. Jay Fruehling (68), Sandra Harding (68), Elaine R. Peskind (22), Eric C. Petrie (22), Gail Li (22), Ansgar J. Furst (60), Steven Chao (60)

Affiliations:

1) UC San Francisco, 2) UC San Diego, 3) Mayo Clinic, Rochester, 4) UC Berkeley, 5) University of Pennsylvania, 6) USC, 7) UC Davis, 8) Brigham and Women's Hospital/Harvard Medical School, 9) Indiana University, 10) Washington University St. Louis, 11) Prevent Alzheimer's Disease 2020, 12) Siemens, 13) Alzheimer's Association, 14) Cornell University, 15) Albert Einstein College of Medicine of Yeshiva University, 16) AD Drug Discovery Foundation, 17) Acumen Pharmaceuticals, 18) Northwestern University, 19) National Institute of Mental Health, 20) Brown University, 21) Eli Lilly, 22) University of Washington, 23) University of London, 24) UCLA School of Medicine, 25) Mayo Clinic, 26) University of Michigan, 27) University of Utah, 28) Banner Alzheimer's Institute, 29) University of Pittsburgh, 30) University of Pennsylvania School of Medicine, 31) UC Irvine, 32) National Institute on Aging, 33) Johns Hopkins University, 34) Richard Frank Consulting, 35) Oregon Health and Science University, 36) Baylor College of Medicine, 37) Columbia University Medical Center, 38) University of Alabama - Birmingham, 39) Mount Sinai School of Medicine, 40) Rush University Medical Center, 41) Wien Center, 42) New York University, 43) Duke University Medical Center, 44) University of Kentucky, 45) University of Rochester Medical Center, 46) University of Texas Southwestern Medical School, 47) Emory University, 48) University of Kansas, Medical Center, 49) UC Los Angeles, 50) Mayo Clinic, Jacksonville, 51) Yale University School of Medicine, 52) McGill Univ., Montreal-Jewish General Hospital, 53) Sunnybrook Health Sciences, Ontario, 54) U.B.C. Clinic for AD & Related Disorders, 55) Cognitive Neurology - St. Joseph's, Ontario, 56) Cleveland Clinic Lou Ruvo Center for Brain Health, 57) Premiere Research Inst (Palm Beach Neurology), 58) Georgetown University Medical Center, 59) Brigham and Women's Hospital, 60) Stanford University, 61) Banner Sun Health Research Institute, 62) Boston University, 63) Howard University, 64) Case Western Reserve University, 65) UC Davis - Sacramento, 66) Neurological Care of CNY, 67) Parkwood Hospital, 68) University of Wisconsin, 69) Dent Neurologic Institute, 70) Ohio State University, 71) Albany Medical College, 72) Hartford Hospital, Olin Neuropsychiatry Research Center, 73) Dartmouth-Hitchcock Medical Center, 74) Wake Forest University Health Sciences, 75) Rhode Island Hospital, 76) Butler Hospital, 77) Medical University South Carolina, 78) St. Joseph's Health Care, 79) Nathan Kline Institute, 80) University of Iowa College of Medicine, 81) University of South Florida: USF Health Byrd Alzheimer's Institute, 82) Rehabilitation Institute of Chicago, Feinberg School of Medicine, Northwestern University, 83) Department of Defense

Supplementary Figure and Table Legends

Supplementary Figure 1. Box plots of SNAP25 expression in each of the 5 data sets. Three probesets were present for measuring SNAP25 expression, and plots are shown for each. Probeset 202508_s_at exclusively targets the 3' UTR region rather than the coding region of the SNAP25 gene, whereas the other two probesets contain probesets matching regions in the exons. Since detection of mRNA based on 3' UTR probesets becomes less accurate for genes that have alternative polyadenylation sites in different tissues,[62] and SNAP25 contains multiple alternative polyadenylation sites in brain different from those in other tissues,[63] the probeset 202508_s_at results may be less reflective of true SNAP25 mRNA expression levels.

Supplementary Figure 2. Overall distribution by gender in the data set of healthy controls used for Figure 3, showing relatively equal distribution.

Supplementary Figure 3. NEUROD6 expression as a function of gender across 4 different tissue types in the data set of healthy controls used for Figure 2.

Supplementary Figure 4. SNAP25-related SNPs are associated with AD specifically in APOE4+ male patients. (a) and (c) Plot showing significantly disease-associated SNPs near SNAP25 in APOE4+ male patients, but not in APOE4+ female patients or APOE4- male patients. SNPs related to SNAP25 in APOE4+ male patients in the LOAD and Cell data sets, respectively, are rs6077693 ($p<0.00029$ in male APOE4+, $p<0.7257$ in female APOE4+, and $p<0.7328$ in male APOE4-) and rs6032806 ($p<0.00043$ in male APOE4+, $p<0.6579$ in female APOE4+, and $p<0.6948$ in male APOE4-). (b) and (d) Propensity plots showing the disease risk (positive values) or protection (negative values) in APOE4+ male patients as a function of a patient's status for each of the top SNAP25 SNPs.

Supplementary Table 1: P-Values and fold changes for the list of 24 genes downregulated in AD consistently across 5 data sets.

Supplementary Table 2: Gender GEO search results

Supplementary Table 3: Patient samples identified to have high NEUROD6 expression from the data set of healthy controls used for Figure 2.

Supplementary Table 4: P-Values for SNAP25 gene expression probe levels in male versus female samples in GSE11882. Bold typeface indicates significant p-values; italicization indicates p-values trending towards significance.

Supplementary Table 5: Summary of the 5 gene expression data sets utilized to identify genes downregulated in AD consistently across multiple data sets.

Supplementary Table 6: DAVID pathway enrichment results for 24 genes.

Supplementary Table 7: Significant SNPs in the region of each of the 24 genes according to given thresholds: (a) distance < 1 Mb up and downstream, p < 1e-2, for whole cohort only and (b) distance < 200 kb up and downstream, p < 5e-4, for each patient subset.

Supplementary Table 8: Lists of genes significantly differentially expressed between AD and control in 4 out of 5 gene expression datasets.

a) Downregulated genes
b) Upregulated genes

Supplementary Table 9: Gender-stratified differential expression analysis.

Supplementary Table 10: Lists of genes targeted by the compounds of interest, based on literature and data mining: (a) NEUROD6-relevant compounds and (b) SNAP25-relevant compounds.

Supplementary Table 11: Differential expression analysis between genders, stratifying by disease status.

Supplementary Table 12: Results of Fisher's exact tests examining potential gender bias in gene expression datasets.

Supplementary Figure 1

SNAP25 — Significant Probesets — Non Significant Probesets

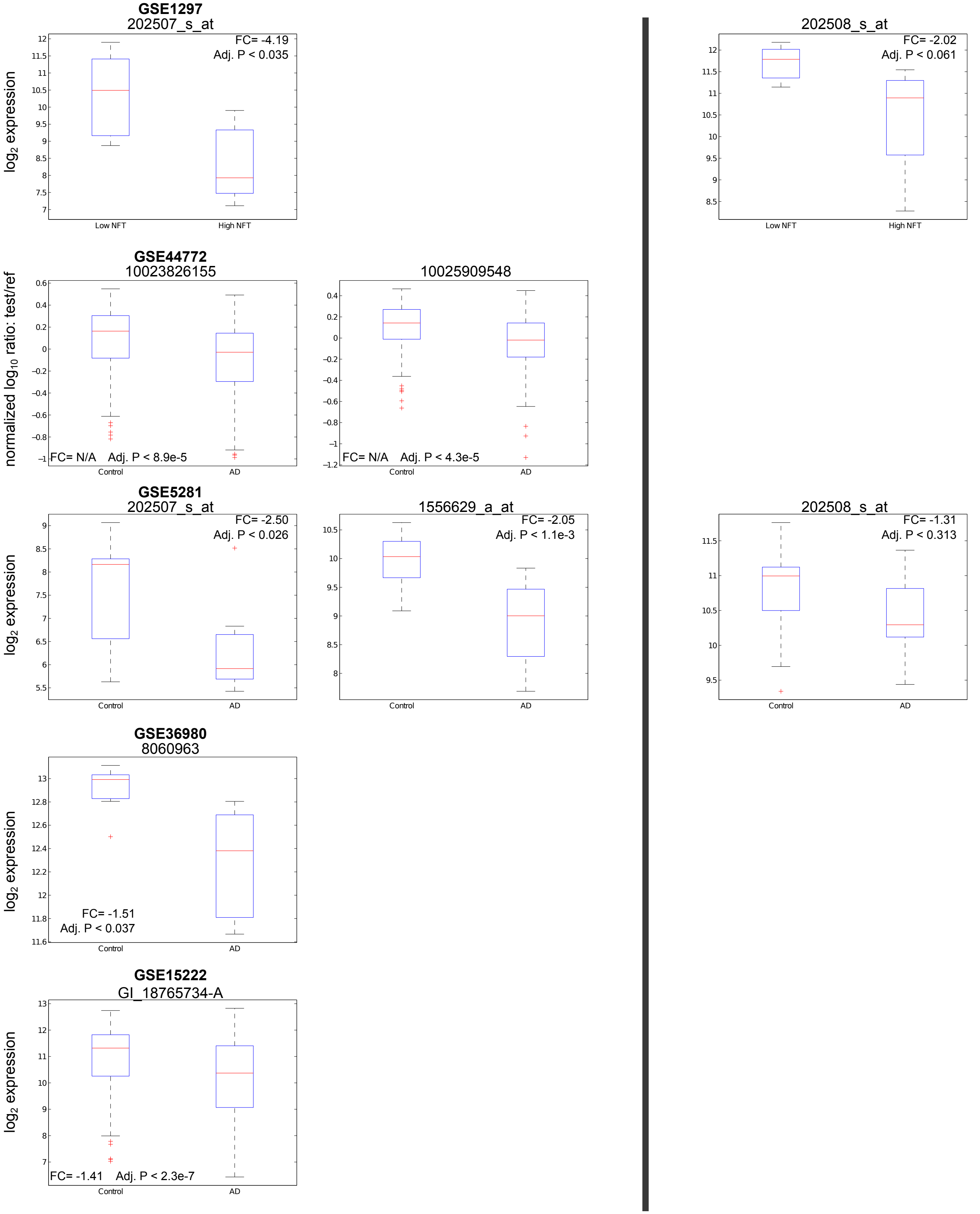

Supplementary Figure 2

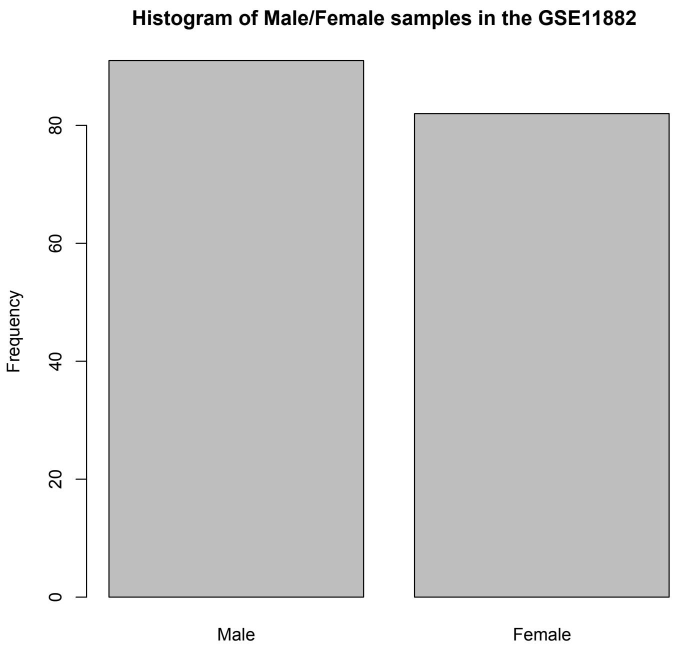

Supplementary Figure 3

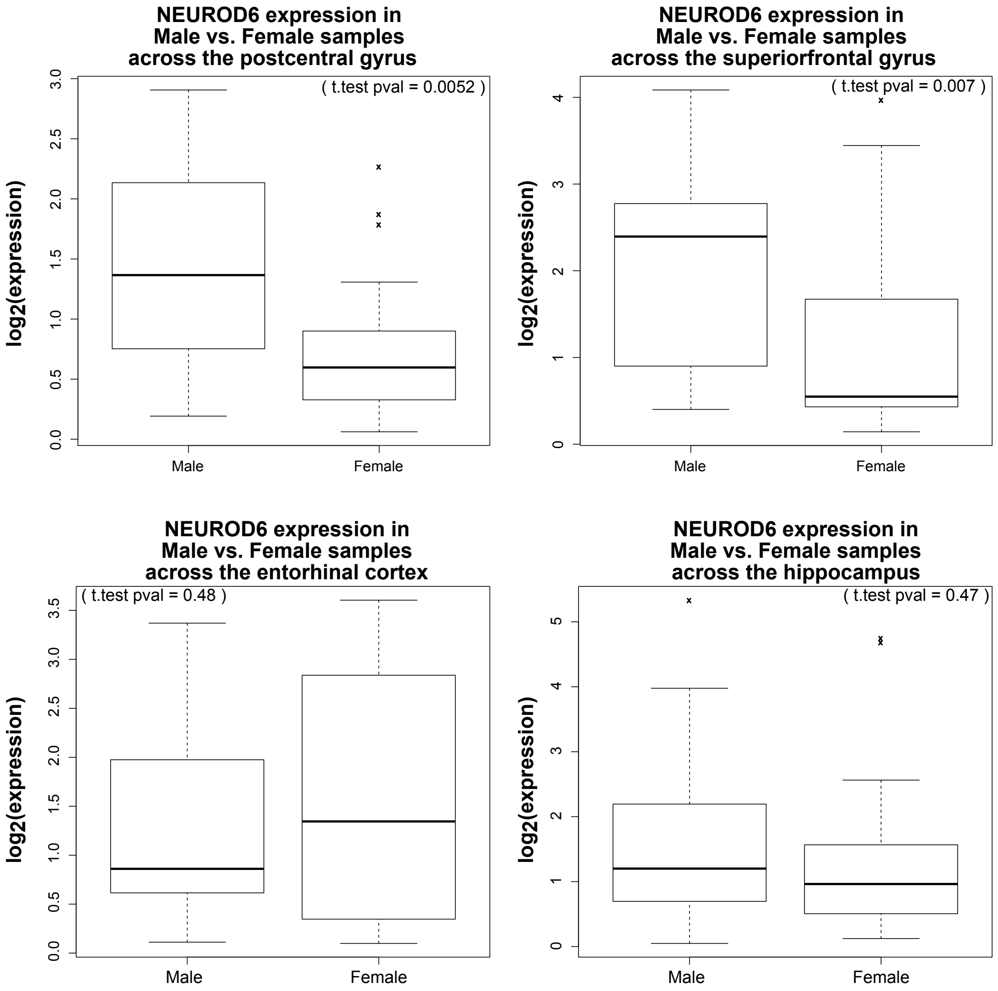

Supplementary Figure 4

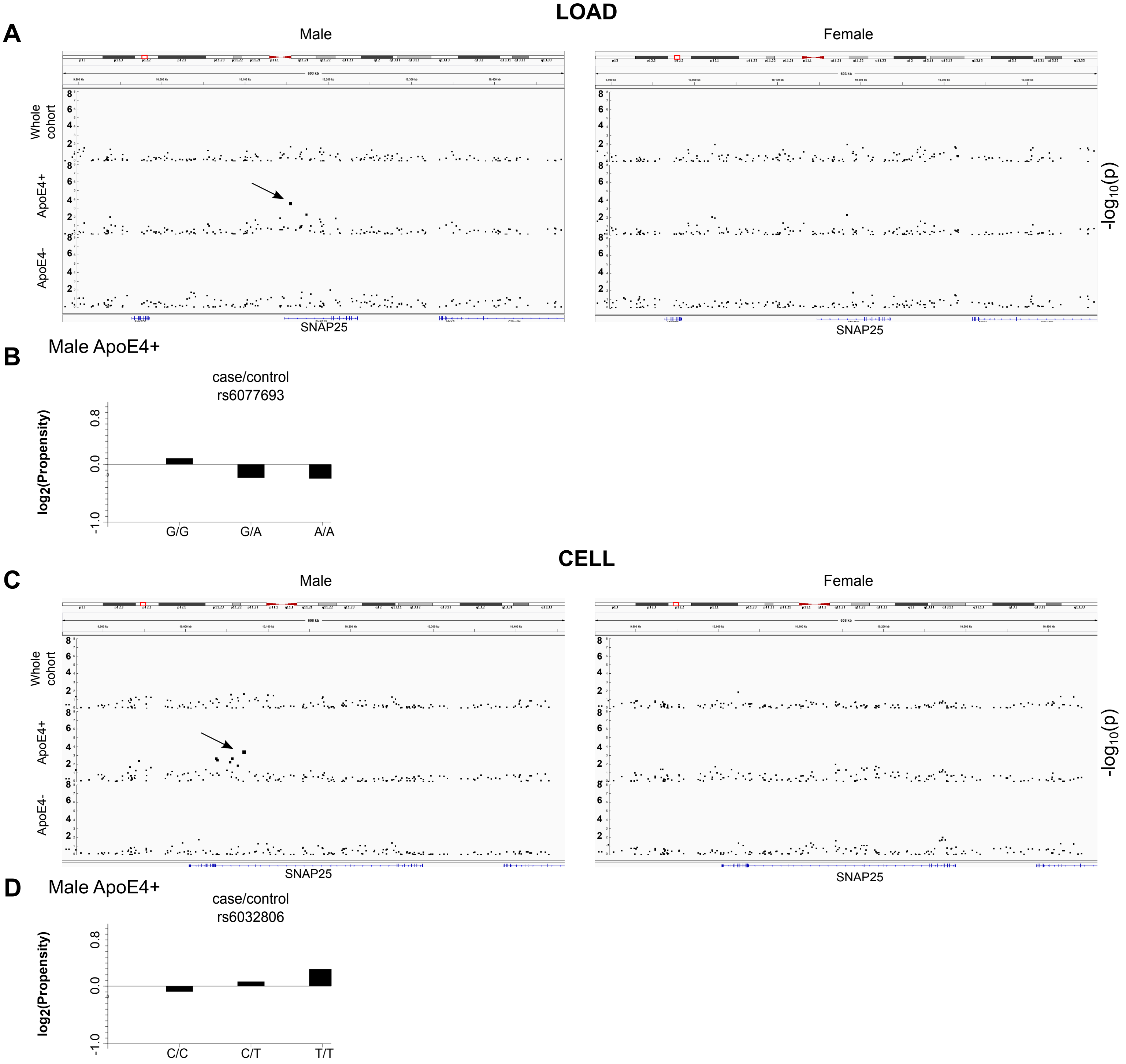

Supplementary Table 1

	GSE5281			GSE1297			GSE36980			GSE15222			GSE44772		
Gene Symbol	FC	P.Value	adj.P.Val	FC	P.Value	adj.P.Val	FC	P.Value	adj.P.Val	FC	P.Value	adj.P.Val	FC	P.Value	adj.P.Val
AP3B2	-2.33	1.08E-05	3.17E-04	-1.79	3.46E-04	3.54E-02	-1.46	3.85E-04	3.65E-02	-1.15	1.73E-06	5.31E-06	NA	2.70E-05	1.26E-04
ATP1A3	-4.36	6.92E-07	5.15E-05	-3.22	5.97E-04	4.09E-02	-1.47	2.65E-04	3.41E-02	-1.19	7.57E-08	2.96E-07	NA	2.77E-08	4.38E-07
ATP5B	-3.13	2.43E-04	2.96E-03	-1.91	5.09E-04	3.95E-02	-1.30	5.96E-04	3.97E-02	-1.14	1.25E-09	6.93E-09	NA	3.11E-06	2.00E-05
ATP6V1E1	-2.26	1.62E-03	1.18E-02	-1.75	6.04E-04	4.09E-02	-1.37	6.88E-05	2.42E-02	-1.36	3.90E-16	1.01E-14	NA	1.33E-07	1.51E-06
ATP6V1G2	-3.56	1.52E-04	2.10E-03	-2.81	1.16E-04	2.91E-02	-1.58	5.71E-05	2.42E-02	-1.33	2.39E-11	1.95E-10	NA	3.41E-06	2.16E-05
BNIP3	-2.04	3.14E-05	6.77E-04	-1.97	8.41E-04	4.56E-02	-1.28	3.10E-04	3.44E-02	-1.13	3.45E-08	1.45E-07	NA	8.90E-07	7.00E-06
C14orf132	-2.12	1.17E-04	1.73E-03	-1.37	4.85E-04	3.86E-02	-1.27	4.24E-04	3.69E-02	-1.08	2.43E-03	4.29E-03	NA	2.22E-07	2.27E-06
C14orf2	-2.27	1.46E-04	2.04E-03	-1.53	3.62E-04	3.57E-02	-1.39	6.63E-04	4.13E-02	-1.42	2.52E-24	1.28E-21	NA	8.28E-07	6.60E-06
CACNG3	-3.35	1.29E-06	7.59E-05	-2.19	1.38E-04	2.98E-02	-1.92	4.62E-04	3.76E-02	-1.55	9.66E-15	1.77E-13	NA	1.88E-06	1.31E-05
GNG3	-4.60	8.67E-06	2.72E-04	-1.90	1.78E-04	3.18E-02	-1.51	7.17E-04	4.19E-02	-1.34	9.46E-19	5.28E-17	NA	1.77E-09	6.16E-08
GOT2	-2.31	7.63E-04	6.77E-03	-1.56	6.62E-05	2.62E-02	-1.37	5.37E-04	3.90E-02	-1.16	5.71E-07	1.90E-06	NA	1.36E-06	9.98E-06
MAGED1	-2.04	1.06E-02	4.61E-02	-1.85	5.20E-04	3.95E-02	-1.29	4.03E-04	3.66E-02	-1.29	1.23E-14	2.19E-13	NA	3.28E-09	9.47E-08
MRPS11	-1.80	6.76E-04	6.19E-03	-1.29	7.00E-04	4.25E-02	-1.27	9.58E-05	2.61E-02	-1.14	9.18E-04	1.75E-03	NA	9.12E-08	1.12E-06
NEUROD6	-1.78	1.67E-05	4.36E-04	-1.74	2.99E-04	3.33E-02	-2.27	2.25E-04	3.29E-02	-2.01	4.18E-25	2.58E-22	NA	3.29E-14	9.22E-11
PPP1R11	-2.83	1.54E-05	4.11E-04	-1.22	2.36E-04	3.32E-02	-1.27	4.71E-06	1.46E-02	-1.11	5.78E-08	2.31E-07	NA	6.04E-05	2.54E-04
PTPRN2	-1.89	6.20E-03	3.14E-02	-2.00	1.64E-04	3.18E-02	-1.43	2.74E-04	3.43E-02	-1.43	7.36E-15	1.38E-13	NA	2.50E-08	4.06E-07
RGS7	-2.65	9.45E-05	1.48E-03	-2.32	1.58E-04	3.17E-02	-1.63	2.33E-04	3.30E-02	-1.39	3.06E-09	1.57E-08	NA	1.54E-08	2.85E-07
SLC17A7	-4.59	2.79E-08	8.14E-06	-2.64	3.00E-04	3.33E-02	-1.54	4.29E-04	3.70E-02	-1.59	1.51E-09	8.22E-09	NA	1.21E-06	9.00E-06
SLC25A11	-1.65	4.39E-04	4.54E-03	-1.58	9.79E-04	4.77E-02	-1.26	1.18E-03	4.88E-02	-1.12	2.37E-06	7.08E-06	NA	4.40E-06	2.66E-05
SNAP25	-2.05	5.94E-05	1.06E-03	-4.19	3.34E-04	3.49E-02	-1.51	4.28E-04	3.70E-02	-1.41	5.75E-08	2.30E-07	NA	7.72E-06	4.30E-05
SYP	-2.87	5.72E-08	1.15E-05	-1.90	2.92E-04	3.33E-02	-1.43	3.03E-04	3.44E-02	-1.47	2.18E-14	3.69E-13	NA	1.83E-04	6.68E-04
TPI1	-3.91	1.27E-06	7.53E-05	-1.62	2.66E-04	3.33E-02	-1.47	7.29E-06	1.46E-02	-1.30	1.80E-06	5.47E-06	NA	1.83E-04	6.68E-04
UQCRC1	-2.58	2.92E-03	1.81E-02	-1.70	7.12E-04	4.25E-02	-1.18	4.12E-04	3.69E-02	-1.11	9.54E-05	2.14E-04	NA	5.20E-08	7.18E-07
YWHAB	-3.53	1.24E-03	9.68E-03	-2.43	4.15E-04	3.74E-02	-1.32	9.69E-04	4.59E-02	-1.08	4.55E-03	7.64E-03	NA	5.83E-06	3.39E-05

Supplementary Table 2

Gene	Number of significant male samples	Total number of significant samples	Total number of male samples in GSE11882	Total number of female samples in GSE11882	Hypergeometric p.value for >= number of significant male samples
AP3B2	0	0	91	82	Number of significant samples is < 15
ATP1A3	8	8	91	82	Number of significant samples is < 15
ATP5B	0	0	91	82	Number of significant samples is < 15
ATP6V1E1	0	0	91	82	Number of significant samples is < 15
ATP6V1G2	3	3	91	82	Number of significant samples is < 15
BNIP3	0	0	91	82	Number of significant samples is < 15
C14orf132	1	1	91	82	Number of significant samples is < 15
C14orf2	0	0	91	82	Number of significant samples is < 15
**CACNG3**	18	20	91	82	2.05E-05
CFL1	0	0	91	82	Number of significant samples is < 15
**GNG3**	23	25	91	82	5.43E-07
GOT2	0	0	91	82	Number of significant samples is < 15
MAGED1	0	0	91	82	Number of significant samples is < 15
MRPS11	0	0	91	82	Number of significant samples is < 15
**NEUROD6**	32	40	91	82	9.76E-06
PPP1R11	0	0	91	82	Number of significant samples is < 15
PTPRN2	0	0	91	82	Number of significant samples is < 15
RGS7	0	0	91	82	Number of significant samples is < 15
SLC17A7	0	0	91	82	Number of significant samples is < 15
SLC25A11	0	0	91	82	Number of significant samples is < 15
SNAP25	0	0	91	82	Number of significant samples is < 15
SYP	13	14	91	82	Number of significant samples is < 15
TPI1	0	0	91	82	Number of significant samples is < 15
UQCRC1	0	0	91	82	Number of significant samples is < 15
YWHAB	0	0	91	82	Number of significant samples is < 15

Supplementary Table 3					
**Sample ID**	**Sample Name**	**Gender**	**Dataset**	**p.val**	**adj.p.val**
GSM300282	SuperiorFrontalGyrus_male_20yrs_indiv78	M	GSE11882	0	0.00E+00
GSM300309	Hippocampus_male_22yrs_indiv85	M	GSE11882	0	0.00E+00
GSM300270	SuperiorFrontalGyrus_male_86yrs_indiv73	M	GSE11882	7.38E-13	8.01E-10
GSM300254	SuperiorFrontalGyrus_male_21yrs_indiv66	M	GSE11882	3.80E-12	3.92E-09
GSM300286	Hippocampus_male_69yrs_indiv8	M	GSE11882	3.80E-12	3.92E-09
GSM300307	SuperiorFrontalGyrus_male_33yrs_indiv84	M	GSE11882	4.38E-12	4.49E-09
GSM300275	EntorhinalCortex_male_20yrs_indiv77	M	GSE11882	1.52E-10	1.45E-07
GSM300246	PostcentralGyrus_male_70yrs_indiv53	M	GSE11882	1.79E-10	1.70E-07
GSM300260	SuperiorFrontalGyrus_male_40yrs_indiv68	M	GSE11882	1.28E-09	1.18E-06
GSM300266	SuperiorFrontalGyrus_male_75yrs_indiv72	M	GSE11882	3.21E-09	2.93E-06
GSM300298	Hippocampus_female_30yrs_indiv82	F	GSE11882	3.37E-09	3.06E-06
GSM300176	SuperiorFrontalGyrus_male_45yrs_indiv12	M	GSE11882	4.07E-09	3.64E-06
GSM300175	PostcentralGyrus_male_45yrs_indiv12	M	GSE11882	3.36E-08	2.89E-05
GSM300339	Hippocampus_female_82yrs_indiv98	F	GSE11882	5.79E-08	4.88E-05
GSM300319	SuperiorFrontalGyrus_male_45yrs_indiv87	M	GSE11882	7.65E-08	6.31E-05
GSM300258	EntorhinalCortex_male_40yrs_indiv68	M	GSE11882	1.86E-07	1.46E-04
GSM300315	SuperiorFrontalGyrus_male_42yrs_indiv86	M	GSE11882	1.94E-07	1.52E-04
GSM300317	Hippocampus_male_45yrs_indiv87	M	GSE11882	2.06E-07	1.61E-04
GSM300264	SuperiorFrontalGyrus_male_52yrs_indiv71	M	GSE11882	4.25E-07	3.21E-04
GSM300235	Hippocampus_male_85yrs_indiv46	M	GSE11882	5.37E-07	3.95E-04
GSM300293	EntorhinalCortex_female_48yrs_indiv81	F	GSE11882	5.86E-07	4.26E-04
GSM300211	SuperiorFrontalGyrus_male_28yrs_indiv29	M	GSE11882	7.03E-07	4.99E-04
GSM300278	SuperiorFrontalGyrus_male_20yrs_indiv77	M	GSE11882	1.21E-06	8.05E-04
GSM300316	EntorhinalCortex_male_45yrs_indiv87	M	GSE11882	1.95E-06	1.20E-03
GSM300281	PostcentralGyrus_male_20yrs_indiv78	M	GSE11882	2.35E-06	1.42E-03
GSM300204	EntorhinalCortex_male_83yrs_indiv28	M	GSE11882	8.78E-06	4.90E-03
GSM300296	SuperiorFrontalGyrus_female_48yrs_indiv81	F	GSE11882	9.29E-06	5.16E-03
GSM300310	PostcentralGyrus_male_22yrs_indiv85	M	GSE11882	9.42E-06	5.22E-03
GSM300206	PostcentralGyrus_male_83yrs_indiv28	M	GSE11882	1.09E-05	5.93E-03
GSM300292	SuperiorFrontalGyrus_female_44yrs_indiv80	F	GSE11882	1.12E-05	6.06E-03
GSM300289	EntorhinalCortex_female_44yrs_indiv80	F	GSE11882	1.19E-05	6.46E-03
GSM300207	SuperiorFrontalGyrus_male_83yrs_indiv28	M	GSE11882	2.57E-05	1.32E-02
GSM300320	EntorhinalCortex_female_47yrs_indiv88	F	GSE11882	3.43E-05	1.69E-02
GSM300269	PostcentralGyrus_male_86yrs_indiv73	M	GSE11882	4.10E-05	1.96E-02
GSM300299	SuperiorFrontalGyrus_female_30yrs_indiv82	F	GSE11882	4.50E-05	2.13E-02
GSM300304	EntorhinalCortex_male_33yrs_indiv84	M	GSE11882	4.84E-05	2.24E-02
GSM300259	PostcentralGyrus_male_40yrs_indiv68	M	GSE11882	4.87E-05	2.26E-02
GSM300303	SuperiorFrontalGyrus_male_20yrs_indiv83	M	GSE11882	6.73E-05	2.97E-02

Supplementary Table 4

	Full Dataset	Entorhinal Cortex	Hippocampus	Postcentral Gyrus	Superiorfrontal Gyrus
202507_a_at	0.13	0.31	0.85	*0.052*	**0.032**
202508_s_at	*0.088*	0.68	0.46	0.25	**0.018**
1556629_a_at	0.2	0.89	0.43	0.23	0.15

Supplementary Table 5					
Dataset	Brain Compartment	Total # of Samples	Array Type	Samples	Notes
GSE1297	Hippocampus	18	Affymetrix Human Genome U133A Array	GSM21204, GSM21206, GSM21207, GSM21209, GSM21211-GSM21213, GSM21216, GSM21218-GSM21222, GSM21224, GSM21226, GSM21230-GSM21232	Compared samples with top 9 NFT scores vs bottom 9 NFT scores
GSE5281	Entorhinal Cortex (Isolated neurons)	23	Affymetrix Human Genome U133 Plus 2.0 Array	GSM119615-GSM119627, GSM238763, GSM238790-GSM238798	Used only the EC samples, compared AD (10 samples) vs healthy (13 samples)
GSE15222	Frontal Cortex, Temporal Cortex, Cerebellum, or Parietal Cortex	364	Sentrix HumanRef-8 Expression BeadChip	All	Compared AD (176 samples) vs healthy (188 samples)
GSE36980	Hippocampus	17	Affymetrix Human Gene 1.0 ST Array	GSM907854-GSM907870	Used only the Hippocampus samples, compared AD (7 samples) vs healthy (10 samples)
GSE44772	Prefrontal Cortex	230	Rosetta/Merck Human 44k 1.1 microarray	GSM1090501-GSM1090730	Used only the Prefrontal Cortex samples, compared AD (129) vs. healthy (101 samples)

Supplementary Table 6

Category	Term	GO_annotation	PValue	Genes	Fold Enrichment	Benjamini
GOTERM_MF_FAT	GO:0015077	monovalent inorganic cation transmembrane transporter activity	1.71E-05	UQCRC1, ATP5B, ATP6V1E1, ATP1A3, ATP6V1G2	29.72298535	0.00233402
GOTERM_MF_FAT	GO:0022890	inorganic cation transmembrane transporter activity	7.37E-05	UQCRC1, ATP5B, ATP6V1E1, ATP1A3, ATP6V1G2	20.47146011	0.00252083
GOTERM_MF_FAT	GO:0015662	ATPase activity, coupled to transmembrane movement of ions, phosphorylative mechanism	6.97E-05	ATP5B, ATP6V1E1, ATP1A3, ATP6V1G2	46.65947889	0.00317918
GOTERM_MF_FAT	GO:0008553	hydrogen-exporting ATPase activity, phosphorylative mechanism	6.28E-05	ATP5B, ATP6V1E1, ATP6V1G2	231.8392857	0.00429127
GOTERM_MF_FAT	GO:0042625	ATPase activity, coupled to transmembrane movement of ions	1.89E-04	ATP5B, ATP6V1E1, ATP1A3, ATP6V1G2	33.41827542	0.00516578
GOTERM_MF_FAT	GO:0015078	hydrogen ion transmembrane transporter activity	3.37E-04	UQCRC1, ATP5B, ATP6V1E1, ATP6V1G2	27.47724868	0.00767082
GOTERM_MF_FAT	GO:0016820	hydrolase activity, acting on acid anhydrides, catalyzing transmembrane movement of substances	6.24E-04	ATP5B, ATP6V1E1, ATP1A3, ATP6V1G2	22.27885028	0.00945131
GOTERM_MF_FAT	GO:0043492	ATPase activity, coupled to movement of substances	6.07E-04	ATP5B, ATP6V1E1, ATP1A3, ATP6V1G2	22.48138528	0.01035074
GOTERM_MF_FAT	GO:0015405	P-P-bond-hydrolysis-driven transmembrane transporter activity	8.21E-04	ATP5B, ATP6V1E1, ATP1A3, ATP6V1G2	20.27010148	0.01119151
GOTERM_MF_FAT	GO:0015399	primary active transmembrane transporter activity	8.21E-04	ATP5B, ATP6V1E1, ATP1A3, ATP6V1G2	20.27010148	0.01119151
GOTERM_MF_FAT	GO:0042626	ATPase activity, coupled to transmembrane movement of substances	5.91E-04	ATP5B, ATP6V1E1, ATP1A3, ATP6V1G2	22.68763652	0.01151127
GOTERM_CC_FAT	GO:0031966	mitochondrial membrane	0.004186103	GOT2, SLC25A11, UQCRC1, ATP5B, BNIP3	7.052527036	0.0327487
GOTERM_CC_FAT	GO:0044429	mitochondrial part	0.002924664	GOT2, SLC25A11, UQCRC1, ATP5B, MRPS11, BNIP3	5.604092072	0.03325067
GOTERM_CC_FAT	GO:0016469	proton-transporting two-sector ATPase complex	0.002677009	ATP5B, ATP6V1E1, ATP6V1G2	37.04927536	0.03347064
GOTERM_CC_FAT	GO:0031410	cytoplasmic vesicle	0.004060816	SLC17A7, SYP, YWHAB, AP3B2, ATP6V1G2, CACNG3	5.193823649	0.03386487
GOTERM_CC_FAT	GO:0030135	coated vesicle	0.002444944	SLC17A7, SYP, AP3B2, ATP6V1G2	13.98085863	0.03395332
GOTERM_CC_FAT	GO:0005740	mitochondrial envelope	0.005209137	GOT2, SLC25A11, UQCRC1, ATP5B, BNIP3	6.631731867	0.03430765
GOTERM_CC_FAT	GO:0044433	cytoplasmic vesicle part	0.003866408	SLC17A7, SYP, ATP6V1G2, CACNG3	11.88746803	0.03453152
GOTERM_CC_FAT	GO:0005886	plasma membrane	0.003712067	PTPRN2, ATP5B, ATP1A3, CACNG3, SLC17A7, SYP, GOT2, MAGED1, SLC25A11, ATP6V1E1, AP3B2, RGS7, GNG3, SNAP25	2.059927939	0.03567944
GOTERM_CC_FAT	GO:0030285	integral to synaptic vesicle membrane	0.005155032	SYP, ATP6V1G2	370.4927536	0.03580882
GOTERM_CC_FAT	GO:0031982	vesicle	0.004873336	SLC17A7, SYP, YWHAB, AP3B2, ATP6V1G2, CACNG3	4.976768332	0.03583774
GOTERM_CC_FAT	GO:0031988	membrane-bounded vesicle	0.002388552	SLC17A7, SYP, YWHAB, AP3B2, ATP6V1G2, CACNG3	5.870483772	0.03725204
GOTERM_CC_FAT	GO:0012506	vesicle membrane	0.002110754	SLC17A7, SYP, ATP6V1G2, CACNG3	14.72156637	0.03761006
GOTERM_CC_FAT	GO:0031090	organelle membrane	3.05E-04	SLC17A7, GOT2, SYP, SLC25A11, UQCRC1, ATP5B, BNIP3, ATP6V1G2, CACNG3	4.563551254	0.03805617
GOTERM_CC_FAT	GO:0030665	clathrin coated vesicle membrane	0.003695299	SLC17A7, SYP, ATP6V1G2	31.45693191	0.03842337
GOTERM_CC_FAT	GO:0016023	cytoplasmic membrane-bounded vesicle	0.002073877	SLC17A7, SYP, YWHAB, AP3B2, ATP6V1G2, CACNG3	6.062608696	0.04299116
GOTERM_CC_FAT	GO:0030662	coated vesicle membrane	0.006902389	SLC17A7, SYP, ATP6V1G2	22.8385944	0.04302895
GOTERM_CC_FAT	GO:0030672	synaptic vesicle membrane	7.00E-04	SLC17A7, SYP, ATP6V1G2	72.48771267	0.04349025
GOTERM_CC_FAT	GO:0008021	synaptic vesicle	0.007462188	SLC17A7, SYP, ATP6V1G2	21.93707094	0.04428707
GOTERM_CC_FAT	GO:0044459	plasma membrane part	0.001956707	SLC17A7, SYP, SLC25A11, PTPRN2, ATP6V1E1, AP3B2, ATP1A3, RGS7, CACNG3, GNG3, SNAP25	2.774911682	0.04853183
GOTERM_CC_FAT	GO:0019717	synaptosome	0.009260472	SLC17A7, SYP, SNAP25	19.61432225	0.05229048
GOTERM_CC_FAT	GO:0005739	mitochondrion	0.00172017	GOT2, SLC25A11, UQCRC1, ATP5B, ATP6V1E1, MRPS11, BNIP3, C14ORF2	4.090076397	0.05319529
GOTERM_CC_FAT	GO:0030659	cytoplasmic vesicle membrane	0.001665714	SLC17A7, SYP, ATP6V1G2, CACNG3	15.99249296	0.06814124
GOTERM_CC_FAT	GO:0005743	mitochondrial inner membrane	0.01493621	GOT2, SLC25A11, UQCRC1, ATP5B	7.264563797	0.07973718
GOTERM_CC_FAT	GO:0031975	envelope	0.020185661	GOT2, SLC25A11, UQCRC1, ATP5B, BNIP3	4.467356354	0.08834448
GOTERM_CC_FAT	GO:0030136	clathrin-coated vesicle	0.021362039	SLC17A7, SYP, ATP6V1G2	12.63043478	0.09023111
GOTERM_CC_FAT	GO:0045202	synapse	0.022117974	SLC17A7, SYP, ATP6V1G2, SNAP25	6.261849357	0.09033945

GOTERM_CC_FAT	GO:0031967	organelle envelope	0.01997198	GOT2, SLC25A11, UQCRC1, ATP5B, BNIP3	4.481767181	0.09052982
GOTERM_CC_FAT	GO:0044455	mitochondrial membrane part	0.01928734	UQCRC1, ATP5B, BNIP3	13.33773913	0.09074676
GOTERM_CC_FAT	GO:0031301	integral to organelle membrane	0.0187113	SYP, BNIP3, ATP6V1G2	13.55461294	0.09149414
GOTERM_CC_FAT	GO:0019866	organelle inner membrane	0.018108355	GOT2, SLC25A11, UQCRC1, ATP5B	6.756706753	0.09217314
GOTERM_MF_FAT	GO:0042623	ATPase activity, coupled	0.007962851	ATP5B, ATP6V1E1, ATP1A3, ATP6V1G2	9.091736695	0.09477401
GOTERM_CC_FAT	GO:0031300	intrinsic to organelle membrane	0.025777294	SYP, BNIP3, ATP6V1G2	11.4192972	0.10146417
GOTERM_CC_FAT	GO:0033178	proton-transporting two-sector ATPase complex, catalytic domain	0.030551975	ATP5B, ATP6V1E1	61.74879227	0.11586369
GOTERM_MF_FAT	GO:0017075	syntaxin-1 binding	0.012260854	SYP, SNAP25	154.5595238	0.13137458
GOTERM_MF_FAT	GO:0016887	ATPase activity	0.013902648	ATP5B, ATP6V1E1, ATP1A3, ATP6V1G2	7.404049045	0.13717252
GOTERM_CC_FAT	GO:0012505	endomembrane system	0.042133686	SLC17A7, SYP, BNIP3, ATP6V1G2, CACNG3	3.553319248	0.15267076
GOTERM_CC_FAT	GO:0005834	heterotrimeric G-protein complex	0.048781792	RGS7, GNG3	38.32683658	0.17039762
GOTERM_CC_FAT	GO:0005759	mitochondrial matrix	0.057471503	GOT2, ATP5B, MRPS11	7.344570006	0.19327462
GOTERM_CC_FAT	GO:0031980	mitochondrial lumen	0.057471503	GOT2, ATP5B, MRPS11	7.344570006	0.19327462
GOTERM_CC_FAT	GO:0044456	synapse part	0.06621624	SLC17A7, SYP, ATP6V1G2	6.777306469	0.21469996
GOTERM_CC_FAT	GO:0019897	extrinsic to plasma membrane	0.082679475	RGS7, GNG3	22.22956522	0.25637112
GOTERM_MF_FAT	GO:0032403	protein complex binding	0.036046555	SYP, UQCRC1, YWHAB	9.462827988	0.2848804
GOTERM_MF_FAT	GO:0046961	proton-transporting ATPase activity, rotational mechanism	0.034866038	ATP5B, ATP6V1E1	53.75983437	0.29339186
GOTERM_MF_FAT	GO:0019905	syntaxin binding	0.046720298	SYP, SNAP25	39.88632873	0.3361435
GOTERM_MF_FAT	GO:0000149	SNARE binding	0.055520081	SYP, SNAP25	33.41827542	0.35257554
GOTERM_MF_FAT	GO:0019829	cation-transporting ATPase activity	0.054058829	ATP5B, ATP6V1E1	34.34656085	0.36101117

Supplementary Table 7a

SNPs present in whole cohort using thresholds of 1 Mb distance, p < 10-2.

Gene	*Cohort*	*Study*	*SNP*	*Chromo-some*	*SNP location*	*Distance from gene*	*Relative to gene*	*GWAS p value*
**AP3B2**								
	whole_cohort	LOAD	rs7168376	15	81814704	844577	downstream	0.005544
	whole_cohort	LOAD	rs11858704	15	81767411	891870	downstream	0.009278
**ATP1A3**								
	whole_cohort	LOAD	rs7260129	19	41922092	44384	downstream	3.23E-05
	whole_cohort	LOAD	rs717225	19	41889214	77262	downstream	3.43E-05
	whole_cohort	LOAD	rs12104228	19	41894376	72100	downstream	0.0001055
	whole_cohort	LOAD	rs1529717	19	41377292	589184	downstream	0.000208
	whole_cohort	LOAD	rs1206032	19	42367143	372867	upstream	0.0009426
	whole_cohort	LOAD	rs8179181	19	41332300	634176	downstream	0.001139
	whole_cohort	LOAD	rs13346092	19	42383303	389027	upstream	0.001388
	whole_cohort	LOAD	rs13346080	19	42383339	389063	upstream	0.002011
	whole_cohort	LOAD	rs3826706	19	42289437	295161	upstream	0.002295
	whole_cohort	CELL	rs2287940	19	41114513	851963	downstream	0.003169
	whole_cohort	LOAD	rs3745938	19	41564812	401664	downstream	0.003891
	whole_cohort	CELL	rs11673698	19	41066469	900007	downstream	0.004541
	whole_cohort	LOAD	rs10405494	19	42897783	903507	upstream	0.006796
	whole_cohort	LOAD	rs10405998	19	42898023	903747	upstream	0.006796
	whole_cohort	CELL	rs12974071	19	41135228	831248	downstream	0.008637
	whole_cohort	LOAD	rs8110922	19	42467169	472893	upstream	0.008991
**ATP5B**								
	whole_cohort	CELL	rs11171660	12	55700032	938143	downstream	0.0002867
	whole_cohort	LOAD	rs12812221	12	56925551	279483	upstream	0.0003633
	whole_cohort	CELL	rs3759097	12	55710018	928157	downstream	0.00054
	whole_cohort	CELL	rs1800974	12	55695572	942603	downstream	0.0007373
	whole_cohort	LOAD	rs2695783	12	56194668	443507	downstream	0.0007703
	whole_cohort	LOAD	rs11832720	12	56863459	217391	upstream	0.001027
	whole_cohort	LOAD	rs3782331	12	56953505	307437	upstream	0.001277
	whole_cohort	LOAD	rs10506347	12	56937302	291234	upstream	0.001666
	whole_cohort	LOAD	rs844469	12	56926025	279957	upstream	0.002536
	whole_cohort	LOAD	rs1072669	12	56948403	302335	upstream	0.00314
	whole_cohort	CELL	rs772254	12	55730547	907628	downstream	0.003159
	whole_cohort	LOAD	rs901069	12	56952594	306526	upstream	0.004669
	whole_cohort	LOAD	rs840160	12	56930711	284643	upstream	0.005396
	whole_cohort	LOAD	rs167769	12	57109991	463923	upstream	0.007372
	whole_cohort	LOAD	rs7136770	12	56987474	341406	upstream	0.007716
	whole_cohort	LOAD	rs4016338	12	57005230	359162	upstream	0.0078
	whole_cohort	LOAD	rs4319546	12	56953043	306975	upstream	0.007976
	whole_cohort	CELL	rs772262	12	55769949	868226	downstream	0.008638
**ATP6V1E1**								
	whole_cohort	LOAD	rs1296794	22	17541914	50222	downstream	5.18E-05
	whole_cohort	LOAD	rs1296792	22	17538826	53310	downstream	6.04E-05
	whole_cohort	LOAD	rs1296793	22	17539250	52886	downstream	6.04E-05
	whole_cohort	LOAD	rs174294	22	17515555	76581	downstream	0.0001572
	whole_cohort	ADNI	rs464385	22	18088241	459419	upstream	0.0001916
	whole_cohort	LOAD	rs5747494	22	18146260	517438	upstream	0.0005574
	whole_cohort	LOAD	rs7287843	22	17611339	0	in gene	0.0006871
	whole_cohort	LOAD	rs2401125	22	17531248	60888	downstream	0.0009838

	whole_cohort	ADNI	rs3827281	22	18101666	472844	upstream	0.001349
	whole_cohort	CELL	rs1867357	22	18024263	395441	upstream	0.001671
	whole_cohort	LOAD	rs738095	22	17773221	144399	upstream	0.001715
	whole_cohort	LOAD	rs9606682	22	17310673	281463	downstream	0.002965
	whole_cohort	ADNI	rs9605028	22	16674093	918043	downstream	0.003841
	whole_cohort	LOAD	rs458888	22	18050667	421845	upstream	0.00409
	whole_cohort	ADNI	rs5747395	22	17888591	259769	upstream	0.004224
	whole_cohort	LOAD	rs8137637	22	16622826	969310	downstream	0.004765
	whole_cohort	LOAD	rs5992146	22	17993174	364352	upstream	0.005751
	whole_cohort	LOAD	rs455758	22	18050568	421746	upstream	0.006294
	whole_cohort	LOAD	rs9605254	22	17314517	277619	downstream	0.006702
	whole_cohort	ADNI	rs467504	22	18100500	471678	upstream	0.007452
	whole_cohort	LOAD	rs9605317	22	17531009	61127	downstream	0.007817
**ATP6V1G2**								
	whole_cohort	LOAD	rs7748472	6	32480985	934137	upstream	4.79E-05
	whole_cohort	LOAD	rs9267873	6	32231574	684726	upstream	7.21E-05
	whole_cohort	LOAD	rs499691	6	32226561	679713	upstream	0.0002559
	whole_cohort	LOAD	rs2736191	6	31593132	46284	upstream	0.0006514
	whole_cohort	LOAD	rs17201046	6	31731795	184947	upstream	0.00068
	whole_cohort	LOAD	rs2073048	6	32367655	820807	upstream	0.0008849
	whole_cohort	CELL	rs9264904	6	31304775	239676	downstream	0.0009065
	whole_cohort	LOAD	rs7770048	6	32366976	820128	upstream	0.0009649
	whole_cohort	ADNI	rs2844457	6	31893363	346515	upstream	0.001057
	whole_cohort	LOAD	rs17209754	6	32474925	928077	upstream	0.001105
	whole_cohort	LOAD	rs17422797	6	32297750	750902	upstream	0.001296
	whole_cohort	LOAD	rs35571839	6	32544835	997987	upstream	0.001383
	whole_cohort	LOAD	rs412657	6	32243307	696459	upstream	0.001607
	whole_cohort	ADNI	rs485774	6	32323176	776328	upstream	0.00168
	whole_cohort	LOAD	rs10484560	6	32330359	783511	upstream	0.00194
	whole_cohort	ADNI	rs9267956	6	32245860	699012	upstream	0.001987
	whole_cohort	LOAD	rs11969759	6	32053352	506504	upstream	0.002157
	whole_cohort	ADNI	rs8111	6	32115397	568549	upstream	0.002177
	whole_cohort	LOAD	rs16870148	6	32431858	885010	upstream	0.00227
	whole_cohort	LOAD	rs377743	6	32243287	696439	upstream	0.002357
	whole_cohort	ADNI	rs9267947	6	32243440	696592	upstream	0.002446
	whole_cohort	LOAD	rs12526151	6	32336307	789459	upstream	0.00277
	whole_cohort	ADNI	rs9268035	6	32259710	712862	upstream	0.002833
	whole_cohort	LOAD	rs17201248	6	31835352	288504	upstream	0.00293
	whole_cohort	LOAD	rs7774197	6	32078497	531649	upstream	0.002931
	whole_cohort	ADNI	rs2763982	6	31904773	357925	upstream	0.003157
	whole_cohort	LOAD	rs1053924	6	32152937	606089	upstream	0.003299
	whole_cohort	ADNI	rs2239689	6	32062506	515658	upstream	0.003337
	whole_cohort	ADNI	rs2228628	6	32121076	574228	upstream	0.003387
	whole_cohort	ADNI	rs2071293	6	32094909	548061	upstream	0.003716
	whole_cohort	LOAD	rs532385	6	32227581	680733	upstream	0.003756
	whole_cohort	ADNI	rs2844697	6	30964531	579920	downstream	0.003768
	whole_cohort	ADNI	rs7766862	6	32065229	518381	upstream	0.00388
	whole_cohort	LOAD	rs2894253	6	32377762	830914	upstream	0.004068
	whole_cohort	ADNI	rs3134945	6	32178714	631866	upstream	0.004094
	whole_cohort	LOAD	rs34330585	6	32422925	876077	upstream	0.004263
	whole_cohort	ADNI	rs3096697	6	32166732	619884	upstream	0.004312
	whole_cohort	ADNI	rs2071295	6	32070922	524074	upstream	0.004349
	whole_cohort	ADNI	rs652888	6	31883456	336608	upstream	0.004383

whole_cohort	ADNI	rs2074488	6	31272653	271798	downstream	0.004573
whole_cohort	ADNI	rs3130284	6	32172709	625861	upstream	0.004613
whole_cohort	ADNI	rs34131062	6	31268632	275819	downstream	0.004743
whole_cohort	LOAD	rs28994870	6	30704979	839472	downstream	0.004999
whole_cohort	ADNI	rs9268384	6	32368808	821960	upstream	0.005104
whole_cohort	ADNI	rs3130347	6	32166878	620030	upstream	0.005107
whole_cohort	ADNI	rs1033500	6	32339604	792756	upstream	0.005218
whole_cohort	ADNI	rs3134947	6	32177427	630579	upstream	0.005218
whole_cohort	ADNI	rs3130573	6	31138490	405961	downstream	0.005278
whole_cohort	ADNI	rs476885	6	32326934	780086	upstream	0.005628
whole_cohort	LOAD	rs4248166	6	32398643	851795	upstream	0.005753
whole_cohort	CELL	rs4122189	6	31200149	344302	downstream	0.005954
whole_cohort	LOAD	rs2269425	6	32155861	609013	upstream	0.00596
whole_cohort	ADNI	rs533885	6	32325374	778526	upstream	0.006005
whole_cohort	ADNI	rs926594	6	32337592	790744	upstream	0.006017
whole_cohort	ADNI	rs9268256	6	32342086	795238	upstream	0.006017
whole_cohort	ADNI	rs9267873	6	32231574	684726	upstream	0.006072
whole_cohort	LOAD	rs16870123	6	32391682	844834	upstream	0.006186
whole_cohort	LOAD	rs2294884	6	32399481	852633	upstream	0.006235
whole_cohort	ADNI	rs537757	6	32300723	753875	upstream	0.006377
whole_cohort	ADNI	rs552339	6	32324937	778089	upstream	0.006377
whole_cohort	ADNI	rs1033498	6	32340091	793243	upstream	0.006377
whole_cohort	ADNI	rs3817982	6	32338484	791636	upstream	0.006377
whole_cohort	ADNI	rs6930681	6	32336253	789405	upstream	0.006377
whole_cohort	ADNI	rs9268326	6	32362375	815527	upstream	0.006377
whole_cohort	ADNI	rs9405090	6	32330594	783746	upstream	0.006377
whole_cohort	ADNI	rs10807100	6	32335290	788442	upstream	0.006377
whole_cohort	LOAD	rs9332730	6	31944231	397383	upstream	0.006462
whole_cohort	LOAD	rs382259	6	32241249	694401	upstream	0.006563
whole_cohort	ADNI	rs17198616	6	31280186	264265	downstream	0.006589
whole_cohort	LOAD	rs805283	6	31690146	143298	upstream	0.00669
whole_cohort	LOAD	rs3094097	6	30666097	878354	downstream	0.006918
whole_cohort	LOAD	rs3094093	6	30711850	832601	downstream	0.006957
whole_cohort	LOAD	rs805267	6	31671979	125131	upstream	0.007027
whole_cohort	LOAD	rs805269	6	31676504	129656	upstream	0.007027
whole_cohort	ADNI	rs9268368	6	32366177	819329	upstream	0.007403
whole_cohort	ADNI	rs9368713	6	32329559	782711	upstream	0.007403
whole_cohort	LOAD	rs805298	6	31653317	106469	upstream	0.007617
whole_cohort	LOAD	rs805300	6	31650789	103941	upstream	0.007617
whole_cohort	LOAD	rs6910390	6	32073843	526995	upstream	0.007698
whole_cohort	ADNI	rs547261	6	32314255	767407	upstream	0.00783
whole_cohort	ADNI	rs9267803	6	32133984	587136	upstream	0.007846
whole_cohort	LOAD	rs375244	6	32223679	676831	upstream	0.007894
whole_cohort	CELL	rs4713420	6	31025789	518662	downstream	0.007901
whole_cohort	ADNI	rs2395114	6	32265769	718921	upstream	0.008101
whole_cohort	LOAD	rs3749952	6	31715379	168531	upstream	0.008134
whole_cohort	LOAD	rs9469040	6	31712096	165248	upstream	0.008186
whole_cohort	LOAD	rs17423649	6	32389355	842507	upstream	0.008236
whole_cohort	ADNI	rs761187	6	32350258	803410	upstream	0.008436
whole_cohort	ADNI	rs565571	6	32298757	751909	upstream	0.008606
whole_cohort	ADNI	rs204999	6	32142201	595353	upstream	0.008699
whole_cohort	ADNI	rs4713505	6	32137223	590375	upstream	0.008933
whole_cohort	LOAD	rs2395150	6	32358267	811419	upstream	0.009173

	whole_cohort	ADNI	rs6928071	6	31463111	81340	downstream	0.009517
	whole_cohort	ADNI	rs2736428	6	31876146	329298	upstream	0.009885
	whole_cohort	ADNI	rs9468907	6	31247319	297132	downstream	0.009964
	whole_cohort	LOAD	rs1265758	6	32355751	808903	upstream	0.009977
**BNIP3**								
	whole_cohort	LOAD	rs3793670	10	132726206	744275	upstream	2.07E-05
	whole_cohort	LOAD	rs7922380	10	132453779	471848	upstream	0.0002257
	whole_cohort	LOAD	rs10765057	10	131227484	740216	downstream	0.0005766
	whole_cohort	LOAD	rs11146074	10	132977775	995844	upstream	0.0006347
	whole_cohort	LOAD	rs12164734	10	131524437	443263	downstream	0.0009565
	whole_cohort	LOAD	rs10870341	10	132846121	864190	upstream	0.001181
	whole_cohort	LOAD	rs12250241	10	131647284	320416	downstream	0.001248
	whole_cohort	LOAD	rs10829966	10	131270019	697681	downstream	0.002471
	whole_cohort	LOAD	rs11146487	10	132738226	756295	upstream	0.002615
	whole_cohort	LOAD	rs7096456	10	132139964	158033	upstream	0.002653
	whole_cohort	LOAD	rs2918118	10	131288550	679150	downstream	0.002953
	whole_cohort	LOAD	rs7078641	10	131327660	640040	downstream	0.004206
	whole_cohort	LOAD	rs2814183	10	132134842	152911	upstream	0.00449
	whole_cohort	LOAD	rs4880336	10	132120000	138069	upstream	0.004669
	whole_cohort	LOAD	rs6482875	10	131301007	666693	downstream	0.00478
	whole_cohort	LOAD	rs4751321	10	131099165	868535	downstream	0.004797
	whole_cohort	LOAD	rs7897741	10	132470370	488439	upstream	0.004963
	whole_cohort	LOAD	rs11017714	10	131094026	873674	downstream	0.005182
	whole_cohort	LOAD	rs4880419	10	132642604	660673	upstream	0.006491
	whole_cohort	ADNI	rs12098358	10	132873263	891332	upstream	0.007349
	whole_cohort	LOAD	rs4382815	10	131350828	616872	downstream	0.007598
	whole_cohort	LOAD	rs12772482	10	132481354	499423	upstream	0.007629
	whole_cohort	LOAD	rs7096228	10	132481635	499704	upstream	0.007755
	whole_cohort	CELL	rs2051113	10	131023999	943701	downstream	0.008091
	whole_cohort	LOAD	rs4880376	10	132441430	459499	upstream	0.008942
	whole_cohort	LOAD	rs2944499	10	131235051	732649	downstream	0.009002
	whole_cohort	CELL	rs12218716	10	132103182	121251	upstream	0.009166
	whole_cohort	CELL	rs1891791	10	131024643	943057	downstream	0.009442
	whole_cohort	LOAD	rs11818345	10	132539115	557184	upstream	0.009547
**C14orf2**								
	whole_cohort	LOAD	rs4983500	14	104579186	657620	upstream	2.74E-05
	whole_cohort	CELL	rs8003023	14	104271724	350158	upstream	9.59E-05
	whole_cohort	LOAD	rs4077378	14	104557174	635608	upstream	0.0002104
	whole_cohort	LOAD	rs3260	14	103013562	898726	downstream	0.0005279
	whole_cohort	LOAD	rs10142229	14	103998471	76905	upstream	0.0006477
	whole_cohort	LOAD	rs11850704	14	103915349	0	in gene	0.0007513
	whole_cohort	CELL	rs4906431	14	104276610	355044	upstream	0.001308
	whole_cohort	LOAD	rs4983582	14	104525133	603567	upstream	0.001358
	whole_cohort	LOAD	rs10138842	14	103450691	461597	downstream	0.001448
	whole_cohort	CELL	rs10143250	14	104257095	335529	upstream	0.001934
	whole_cohort	LOAD	rs4983437	14	104571087	649521	upstream	0.002894
	whole_cohort	LOAD	rs11848341	14	103523688	388600	downstream	0.003351
	whole_cohort	LOAD	rs7147000	14	104541477	619911	upstream	0.003463
	whole_cohort	ADNI	rs1557012	14	104114734	193168	upstream	0.003774
	whole_cohort	LOAD	rs4548807	14	104574650	653084	upstream	0.004056
	whole_cohort	LOAD	rs8012258	14	104524790	603224	upstream	0.005692
	whole_cohort	LOAD	rs4983599	14	104545098	623532	upstream	0.005872
	whole_cohort	CELL	rs8016595	14	104255188	333622	upstream	0.006675

	whole_cohort	LOAD	rs11851727	14	103356574	555714	downstream	0.006782
	whole_cohort	LOAD	rs11845930	14	103288035	624253	downstream	0.006823
	whole_cohort	CELL	rs7147000	14	104541477	619911	upstream	0.007642
	whole_cohort	LOAD	rs2497295	14	104185059	263493	upstream	0.008573
	whole_cohort	LOAD	rs7157172	14	104532358	610792	upstream	0.008651
	whole_cohort	CELL	rs7157813	14	102997223	915065	downstream	0.00925
**C14orf132**								
	whole_cohort	LOAD	rs17214407	14	96634901	540930	downstream	4.28E-06
	whole_cohort	LOAD	rs10140933	14	96614985	521014	downstream	1.03E-05
	whole_cohort	LOAD	rs10498647	14	95915004	124320	upstream	7.07E-05
	whole_cohort	LOAD	rs17092380	14	95454044	585280	upstream	0.0001435
	whole_cohort	LOAD	rs4578582	14	95627355	411969	upstream	0.0001679
	whole_cohort	LOAD	rs4905510	14	96627321	533350	downstream	0.0005036
	whole_cohort	LOAD	rs2887399	14	95714357	324967	upstream	0.0008417
	whole_cohort	CELL	rs17784096	14	95362646	676678	upstream	0.0008644
	whole_cohort	LOAD	rs10131341	14	95716278	323046	upstream	0.0009736
	whole_cohort	CELL	rs10140933	14	96614985	521014	downstream	0.001487
	whole_cohort	LOAD	rs2369049	14	95705513	333811	upstream	0.00203
	whole_cohort	LOAD	rs942328	14	95356740	682584	upstream	0.002115
	whole_cohort	LOAD	rs9671369	14	95439983	599341	upstream	0.002201
	whole_cohort	LOAD	rs2147606	14	95760861	278463	upstream	0.002355
	whole_cohort	LOAD	rs17280373	14	96669749	575778	downstream	0.002643
	whole_cohort	LOAD	rs8022742	14	96997257	903286	downstream	0.002807
	whole_cohort	CELL	rs8017312	14	95364158	675166	upstream	0.00311
	whole_cohort	ADNI	rs17214365	14	96616880	522909	downstream	0.003648
	whole_cohort	LOAD	rs17094001	14	96292718	198747	downstream	0.003738
	whole_cohort	LOAD	rs1884064	14	96957531	863560	downstream	0.003793
	whole_cohort	ADNI	rs11850274	14	96173160	79189	downstream	0.004048
	whole_cohort	LOAD	rs17094014	14	96317126	223155	downstream	0.004515
	whole_cohort	LOAD	rs10131762	14	96792183	698212	downstream	0.004589
	whole_cohort	LOAD	rs12435173	14	96628606	534635	downstream	0.004961
	whole_cohort	CELL	rs10131762	14	96792183	698212	downstream	0.005403
	whole_cohort	LOAD	rs744439	14	95741452	297872	upstream	0.005509
	whole_cohort	LOAD	rs12882788	14	95204580	834744	upstream	0.005828
	whole_cohort	LOAD	rs7156281	14	95506762	532562	upstream	0.006241
	whole_cohort	CELL	rs10135704	14	96865280	771309	downstream	0.006762
	whole_cohort	ADNI	rs4900324	14	96408649	314678	downstream	0.007019
	whole_cohort	ADNI	rs10873472	14	96463805	369834	downstream	0.007019
	whole_cohort	LOAD	rs12896363	14	95766264	273060	upstream	0.007022
	whole_cohort	LOAD	rs8019857	14	95105737	933587	upstream	0.007768
	whole_cohort	LOAD	rs17214365	14	96616880	522909	downstream	0.007837
	whole_cohort	LOAD	rs12891967	14	95048717	990607	upstream	0.008198
	whole_cohort	LOAD	rs234577	14	96633288	539317	downstream	0.008207
	whole_cohort	CELL	rs2182027	14	95799514	239810	upstream	0.008356
	whole_cohort	LOAD	rs7159713	14	95703239	336085	upstream	0.00865
	whole_cohort	LOAD	rs1951980	14	95786689	252635	upstream	0.008862
	whole_cohort	LOAD	rs9944047	14	96760485	666514	downstream	0.009939
**CACNG3**								
	whole_cohort	LOAD	rs8045868	16	24953196	590780	downstream	1.46E-06
	whole_cohort	LOAD	rs7199880	16	24413065	50649	downstream	0.0001467
	whole_cohort	LOAD	rs8054276	16	25318507	956091	downstream	0.0001503
	whole_cohort	LOAD	rs802758	16	24659231	296815	downstream	0.0004177
	whole_cohort	LOAD	rs7205273	16	23313527	942026	upstream	0.0005004

	whole_cohort	LOAD	rs17256755	16	23325043	930510	upstream	0.0005096
	whole_cohort	LOAD	rs6497739	16	24416541	54125	downstream	0.0005398
	whole_cohort	LOAD	rs11074621	16	24438405	75989	downstream	0.0007476
	whole_cohort	LOAD	rs4787676	16	24045564	209989	upstream	0.0009375
	whole_cohort	LOAD	rs12447134	16	23339347	916206	upstream	0.001302
	whole_cohort	LOAD	rs7191803	16	24422020	59604	downstream	0.00153
	whole_cohort	LOAD	rs7196594	16	24379383	16967	downstream	0.001981
	whole_cohort	LOAD	rs1267577	16	24428724	66308	downstream	0.003032
	whole_cohort	LOAD	rs17841809	16	23324744	930809	upstream	0.004312
	whole_cohort	LOAD	rs16940016	16	23311123	944430	upstream	0.005022
	whole_cohort	LOAD	rs2887481	16	23296593	958960	upstream	0.005671
	whole_cohort	LOAD	rs3815951	16	24943401	580985	downstream	0.005738
	whole_cohort	LOAD	rs4787664	16	24039917	215636	upstream	0.0061
	whole_cohort	LOAD	rs8045616	16	25070962	708546	downstream	0.006125
	whole_cohort	ADNI	rs271005	16	24466083	103667	downstream	0.007353
	whole_cohort	LOAD	rs16974249	16	24950247	587831	downstream	0.008917
	whole_cohort	LOAD	rs274073	16	24876793	514377	downstream	0.009063
	whole_cohort	ADNI	rs6497753	16	24699368	336952	downstream	0.009635
	whole_cohort	LOAD	rs4238911	16	24965819	603403	downstream	0.009637
	whole_cohort	LOAD	rs1011078	16	24882355	519939	downstream	0.009795
**GNG3**								
	whole_cohort	LOAD	rs4963235	11	63051701	342495	downstream	0.0003092
	whole_cohort	LOAD	rs2730034	11	62809760	100554	downstream	0.000427
	whole_cohort	LOAD	rs7109608	11	62436403	270284	upstream	0.0004876
	whole_cohort	LOAD	rs2509978	11	62437310	269377	upstream	0.0006039
	whole_cohort	LOAD	rs1293035	11	62479123	227564	upstream	0.0009183
	whole_cohort	LOAD	rs198475	11	61758598	948089	upstream	0.000968
	whole_cohort	LOAD	rs679626	11	62773759	64553	downstream	0.001043
	whole_cohort	LOAD	rs13377501	11	62404701	301986	upstream	0.00125
	whole_cohort	LOAD	rs10897300	11	62858313	149107	downstream	0.001653
	whole_cohort	LOAD	rs953894	11	62994949	285743	downstream	0.001972
	whole_cohort	LOAD	rs4149183	11	62998396	289190	downstream	0.002071
	whole_cohort	LOAD	rs7113486	11	63004633	295427	downstream	0.00212
	whole_cohort	LOAD	rs12287151	11	63425038	715832	downstream	0.002201
	whole_cohort	LOAD	rs4454725	11	62474595	232092	upstream	0.002234
	whole_cohort	LOAD	rs4149182	11	63000640	291434	downstream	0.002309
	whole_cohort	LOAD	rs17158280	11	63416358	707152	downstream	0.00252
	whole_cohort	LOAD	rs11231242	11	62836443	127237	downstream	0.003096
	whole_cohort	LOAD	rs11231168	11	62634147	72540	upstream	0.004997
	whole_cohort	LOAD	rs1938677	11	62929478	220272	downstream	0.005317
	whole_cohort	ADNI	rs4149181	11	63014448	305242	downstream	0.005546
	whole_cohort	CELL	rs12364908	11	63134058	424852	downstream	0.005679
	whole_cohort	LOAD	rs10750999	11	63631677	922471	downstream	0.007333
	whole_cohort	LOAD	rs259885	11	62049727	656960	upstream	0.007725
	whole_cohort	LOAD	rs11605965	11	62386688	319999	upstream	0.007797
	whole_cohort	CELL	rs10897369	11	63124411	415205	downstream	0.008863
	whole_cohort	LOAD	rs2276299	11	62998958	289752	downstream	0.009613
	whole_cohort	LOAD	rs7925016	11	62276456	430231	upstream	0.009623
**GOT2**								
	whole_cohort	LOAD	rs6499977	16	58773637	39280	upstream	3.11E-06
	whole_cohort	LOAD	rs1482243	16	59571745	837388	upstream	1.27E-05
	whole_cohort	LOAD	rs7201899	16	58677565	29566	downstream	1.74E-05
	whole_cohort	LOAD	rs9936381	16	59575887	841530	upstream	1.93E-05

	whole_cohort	LOAD	rs8056271		16	59553660	819303	upstream	6.72E-05
	whole_cohort	LOAD	rs9934111		16	59609223	874866	upstream	7.41E-05
	whole_cohort	LOAD	rs17796496		16	59572367	838010	upstream	8.62E-05
	whole_cohort	LOAD	rs2202010		16	59612292	877935	upstream	0.0001333
	whole_cohort	LOAD	rs6499969		16	58700302	6829	downstream	0.0001352
	whole_cohort	LOAD	rs1482246		16	59558020	823663	upstream	0.0001678
	whole_cohort	LOAD	rs8049463		16	57913275	793856	downstream	0.00022
	whole_cohort	LOAD	rs6500030		16	59276837	542480	upstream	0.000242
	whole_cohort	LOAD	rs1476307		16	59620524	886167	upstream	0.0003988
	whole_cohort	LOAD	rs247037		16	57889043	818088	downstream	0.0004047
	whole_cohort	LOAD	rs4784044		16	58479908	227223	downstream	0.0004342
	whole_cohort	CELL	rs1437176		16	58922575	188218	upstream	0.0004375
	whole_cohort	LOAD	rs7206052		16	58769606	35249	upstream	0.0006862
	whole_cohort	LOAD	rs423114		16	59641982	907625	upstream	0.0007986
	whole_cohort	LOAD	rs2081248		16	57813971	893160	downstream	0.0009422
	whole_cohort	LOAD	rs1500279		16	59693876	959519	upstream	0.001034
	whole_cohort	LOAD	rs1500278		16	59693619	959262	upstream	0.001042
	whole_cohort	LOAD	rs981666		16	59692376	958019	upstream	0.001097
	whole_cohort	LOAD	rs7203959		16	59252907	518550	upstream	0.001249
	whole_cohort	LOAD	rs9922949		16	57921708	785423	downstream	0.001734
	whole_cohort	CELL	rs2033250		16	57887211	819920	downstream	0.002169
	whole_cohort	LOAD	rs7198505		16	57747851	959280	downstream	0.002173
	whole_cohort	LOAD	rs9888768		16	59552250	817893	upstream	0.002596
	whole_cohort	LOAD	rs8062792		16	58800314	65957	upstream	0.002812
	whole_cohort	LOAD	rs2897521		16	59687159	952802	upstream	0.002937
	whole_cohort	LOAD	rs4468605		16	58055119	652012	downstream	0.00393
	whole_cohort	LOAD	rs6500024		16	59199273	464916	upstream	0.004281
	whole_cohort	LOAD	rs7194216		16	58770476	36119	upstream	0.004339
	whole_cohort	LOAD	rs7192390		16	59553069	818712	upstream	0.004355
	whole_cohort	CELL	rs11640898		16	58880159	145802	upstream	0.004618
	whole_cohort	CELL	rs11646347		16	58879945	145588	upstream	0.004618
	whole_cohort	LOAD	rs1117306		16	59287921	553564	upstream	0.004722
	whole_cohort	LOAD	rs9923128		16	58670061	37070	downstream	0.004889
	whole_cohort	LOAD	rs6500038		16	59505278	770921	upstream	0.00508
	whole_cohort	CELL	rs12599248		16	58871499	137142	upstream	0.005421
	whole_cohort	ADNI	rs2202950		16	59636251	901894	upstream	0.005679
	whole_cohort	LOAD	rs716802		16	58861934	127577	upstream	0.005932
	whole_cohort	LOAD	rs950844		16	58587137	119994	downstream	0.006666
	whole_cohort	LOAD	rs7185424		16	58787630	53273	upstream	0.006761
	whole_cohort	LOAD	rs17185953		16	59319489	585132	upstream	0.007065
	whole_cohort	CELL	rs2059265		16	57881800	825331	downstream	0.007216
	whole_cohort	LOAD	rs8049653		16	58767430	33073	upstream	0.007316
	whole_cohort	CELL	rs247032		16	57878774	828357	downstream	0.00776
	whole_cohort	LOAD	rs2967154		16	57746243	960888	downstream	0.008112
	whole_cohort	LOAD	rs16960332		16	58634939	72192	downstream	0.008722
	whole_cohort	LOAD	rs29861		16	59358242	623885	upstream	0.009734
**MAGED1**									
	whole_cohort	LOAD	rs3897937	X		50912015	891044	upstream	4.03E-05
	whole_cohort	LOAD	rs14115	X		51923689	21335	downstream	0.0001216
	whole_cohort	LOAD	rs5915399	X		51055811	747248	upstream	0.0001683
	whole_cohort	LOAD	rs6521901	X		50935288	867771	upstream	0.000633
	whole_cohort	LOAD	rs4826666	X		51038005	765054	upstream	0.000725
	whole_cohort	LOAD	rs5961263	X		51078992	724067	upstream	0.001112

	whole_cohort	LOAD	rs5915325	X	50835228	967831	upstream	0.001813
	whole_cohort	LOAD	rs5961210	X	50829467	973592	upstream	0.002662
	whole_cohort	ADNI	rs952422	X	50901073	901986	upstream	0.004347
	whole_cohort	LOAD	rs5915196	X	50847555	955504	upstream	0.006
	whole_cohort	ADNI	rs2680826	X	51272979	530080	upstream	0.006806
	whole_cohort	LOAD	rs7889634	X	51166560	636499	upstream	0.00747
	whole_cohort	CELL	rs6645310	X	52276390	374036	downstream	0.008603
	whole_cohort	LOAD	rs7065618	X	50918587	884472	upstream	0.009335
	whole_cohort	LOAD	rs6568280	X	52651550	749196	downstream	0.009375
**MRPS11**								
	whole_cohort	LOAD	rs3743473	15	88630816	152186	downstream	4.37E-06
	whole_cohort	LOAD	rs6496484	15	88442442	25011	upstream	5.88E-06
	whole_cohort	LOAD	rs8027765	15	88626626	147996	downstream	7.34E-06
	whole_cohort	LOAD	rs7172522	15	88636787	158157	downstream	0.0001045
	whole_cohort	LOAD	rs7176734	15	88472054	0	in gene	0.0001258
	whole_cohort	LOAD	rs9920906	15	88471117	0	in gene	0.0003131
	whole_cohort	LOAD	rs11638243	15	88636444	157814	downstream	0.0003849
	whole_cohort	LOAD	rs8042040	15	88384960	82493	upstream	0.00059
	whole_cohort	LOAD	rs6496475	15	88355437	112016	upstream	0.0007102
	whole_cohort	LOAD	rs1126823	15	88874006	395376	downstream	0.0007107
	whole_cohort	LOAD	rs8030854	15	88590317	111687	downstream	0.0007499
	whole_cohort	LOAD	rs11073790	15	88567603	88973	downstream	0.0007877
	whole_cohort	LOAD	rs8037291	15	87964699	502754	upstream	0.0009567
	whole_cohort	CELL	rs7168941	15	89255745	777115	downstream	0.0013
	whole_cohort	LOAD	rs2159082	15	89176819	698189	downstream	0.001583
	whole_cohort	LOAD	rs6496448	15	87856185	611268	upstream	0.001981
	whole_cohort	LOAD	rs8031741	15	88875637	397007	downstream	0.002113
	whole_cohort	LOAD	rs1442291	15	88381490	85963	upstream	0.002192
	whole_cohort	LOAD	rs11857753	15	88204534	262919	upstream	0.00272
	whole_cohort	LOAD	rs8035265	15	88051207	416246	upstream	0.003997
	whole_cohort	LOAD	rs7179950	15	88430501	36952	upstream	0.004377
	whole_cohort	CELL	rs12910780	15	88161880	305573	upstream	0.004718
	whole_cohort	LOAD	rs8030107	15	88004058	463395	upstream	0.005449
	whole_cohort	LOAD	rs8038245	15	87959310	508143	upstream	0.0056
	whole_cohort	LOAD	rs938613	15	88834541	355911	downstream	0.005996
	whole_cohort	LOAD	rs8036142	15	88457265	10188	upstream	0.006563
	whole_cohort	LOAD	rs16941103	15	87982719	484734	upstream	0.006798
	whole_cohort	LOAD	rs8037001	15	89022254	543624	downstream	0.006877
	whole_cohort	CELL	rs16941252	15	88094774	372679	upstream	0.007301
	whole_cohort	ADNI	rs1075725	15	87565084	902369	upstream	0.007439
	whole_cohort	LOAD	rs7170976	15	87964100	503353	upstream	0.007585
	whole_cohort	LOAD	rs8035336	15	88792137	313507	downstream	0.007655
	whole_cohort	LOAD	rs4932432	15	88829369	350739	downstream	0.007984
	whole_cohort	LOAD	rs2289439	15	89142554	663924	downstream	0.00912
	whole_cohort	ADNI	rs12910780	15	88161880	305573	upstream	0.009632
	whole_cohort	CELL	rs4932367	15	88627144	148514	downstream	0.009697
	whole_cohort	LOAD	rs2009966	15	87997492	469961	upstream	0.009914
**NEUROD6**								
	whole_cohort	LOAD	rs10486499	7	32037468	696544	upstream	5.64E-05
	whole_cohort	LOAD	rs6972352	7	31495240	154316	upstream	0.0002405
	whole_cohort	LOAD	rs7803859	7	31166146	171315	downstream	0.001026
	whole_cohort	LOAD	rs10224530	7	31025777	311684	downstream	0.001341
	whole_cohort	LOAD	rs17159410	7	30740108	597353	downstream	0.001341

	whole_cohort	ADNI	rs13231468	7	31367826	26902	upstream	0.002124
	whole_cohort	LOAD	rs978821	7	31882528	541604	upstream	0.002338
	whole_cohort	LOAD	rs7776608	7	30382779	954682	downstream	0.002399
	whole_cohort	LOAD	rs2267716	7	30677026	660435	downstream	0.002747
	whole_cohort	LOAD	rs10227576	7	32316877	975953	upstream	0.002828
	whole_cohort	ADNI	rs10247918	7	31814780	473856	upstream	0.003304
	whole_cohort	ADNI	rs1203181	7	30891691	445770	downstream	0.003796
	whole_cohort	CELL	rs10265249	7	30945960	391501	downstream	0.003992
	whole_cohort	ADNI	rs2267732	7	31094702	242759	downstream	0.004141
	whole_cohort	LOAD	rs10243603	7	31548003	207079	upstream	0.004435
	whole_cohort	LOAD	rs17425178	7	32158533	817609	upstream	0.004553
	whole_cohort	ADNI	rs38402	7	30487746	849715	downstream	0.004664
	whole_cohort	ADNI	rs7805350	7	30889858	447603	downstream	0.004742
	whole_cohort	CELL	rs7780365	7	31398995	58071	upstream	0.005047
	whole_cohort	LOAD	rs6966354	7	30748181	589280	downstream	0.005257
	whole_cohort	LOAD	rs1917011	7	31274254	63207	downstream	0.005958
	whole_cohort	LOAD	rs38402	7	30487746	849715	downstream	0.006066
	whole_cohort	ADNI	rs2107552	7	30821088	516373	downstream	0.006085
	whole_cohort	LOAD	rs2267732	7	31094702	242759	downstream	0.006609
	whole_cohort	LOAD	rs13242518	7	31945365	604441	upstream	0.006945
	whole_cohort	LOAD	rs7794213	7	31477775	136851	upstream	0.007879
	whole_cohort	LOAD	rs7783525	7	31324078	13383	downstream	0.008487
	whole_cohort	LOAD	rs10236961	7	31034598	302863	downstream	0.00867
	whole_cohort	ADNI	rs10258097	7	30836815	500646	downstream	0.009366
	whole_cohort	ADNI	rs10951282	7	31178195	159266	downstream	0.009549
	whole_cohort	LOAD	rs12701070	7	31250840	86621	downstream	0.009927
**PPP1R11**								
	whole_cohort	LOAD	rs238883	6	29378448	688246	upstream	1.29E-05
	whole_cohort	LOAD	rs3129110	6	29117450	949244	upstream	3.05E-05
	whole_cohort	LOAD	rs3130778	6	29131801	934893	upstream	4.77E-05
	whole_cohort	LOAD	rs4711185	6	29388268	678426	upstream	0.0001702
	whole_cohort	LOAD	rs9295805	6	29430001	636693	upstream	0.0001725
	whole_cohort	LOAD	rs9405124	6	29401035	665659	upstream	0.0001725
	whole_cohort	LOAD	rs2142905	6	29096994	969700	upstream	0.0002468
	whole_cohort	LOAD	rs1003581	6	29572426	494268	upstream	0.0003379
	whole_cohort	CELL	rs2074483	6	30108977	38644	downstream	0.0006436
	whole_cohort	LOAD	rs3130718	6	29146843	919851	upstream	0.0007376
	whole_cohort	LOAD	rs3130729	6	29157969	908725	upstream	0.0007376
	whole_cohort	LOAD	rs238882	6	29378585	688109	upstream	0.0007561
	whole_cohort	LOAD	rs909968	6	29531727	534967	upstream	0.0007785
	whole_cohort	LOAD	rs3116847	6	29151035	915659	upstream	0.000985
	whole_cohort	CELL	rs2747442	6	29685408	381286	upstream	0.00105
	whole_cohort	LOAD	rs2206041	6	29210828	855866	upstream	0.001084
	whole_cohort	LOAD	rs2206040	6	29210736	855958	upstream	0.001091
	whole_cohort	LOAD	rs446198	6	29539648	527046	upstream	0.001153
	whole_cohort	CELL	rs3129054	6	29681278	385416	upstream	0.001235
	whole_cohort	ADNI	rs3025652	6	29571987	494707	upstream	0.001253
	whole_cohort	LOAD	rs3131084	6	29074894	991800	upstream	0.00139
	whole_cohort	LOAD	rs376681	6	29549315	517379	upstream	0.001508
	whole_cohort	CELL	rs3117292	6	29684421	382273	upstream	0.00152
	whole_cohort	ADNI	rs1362076	6	29473391	593303	upstream	0.001648
	whole_cohort	LOAD	rs6903989	6	29334252	732442	upstream	0.001722
	whole_cohort	ADNI	rs1003581	6	29572426	494268	upstream	0.001885

whole_cohort	CELL	rs1611285	6	29764009	302685	upstream	0.001985
whole_cohort	LOAD	rs10456370	6	29312958	753736	upstream	0.002
whole_cohort	CELL	rs9257936	6	29671998	394696	upstream	0.002026
whole_cohort	CELL	rs9261154	6	30023159	43535	upstream	0.00213
whole_cohort	LOAD	rs3025652	6	29571987	494707	upstream	0.002153
whole_cohort	CELL	rs5025708	6	29987421	79273	upstream	0.00216
whole_cohort	LOAD	rs17195362	6	30490650	420317	downstream	0.002211
whole_cohort	LOAD	rs4598109	6	29376926	689768	upstream	0.002465
whole_cohort	LOAD	rs7763162	6	30179578	109245	downstream	0.002492
whole_cohort	LOAD	rs3132674	6	30234651	164318	downstream	0.002506
whole_cohort	CELL	rs2474102	6	29682863	383831	upstream	0.002513
whole_cohort	CELL	rs2517830	6	29861788	204906	upstream	0.002639
whole_cohort	CELL	rs9261564	6	30206812	136479	downstream	0.002746
whole_cohort	CELL	rs2517938	6	29742062	324632	upstream	0.002792
whole_cohort	CELL	rs2747454	6	29687335	379359	upstream	0.002874
whole_cohort	CELL	rs3734838	6	30112453	42120	downstream	0.002874
whole_cohort	LOAD	rs2076483	6	29603767	462927	upstream	0.003035
whole_cohort	LOAD	rs2267633	6	29603063	463631	upstream	0.003035
whole_cohort	LOAD	rs3129105	6	29245292	821402	upstream	0.003138
whole_cohort	CELL	rs2394160	6	29735484	331210	upstream	0.00317
whole_cohort	LOAD	rs6457164	6	30187330	116997	downstream	0.003317
whole_cohort	LOAD	rs388234	6	29565517	501177	upstream	0.003514
whole_cohort	LOAD	rs2106070	6	30219085	148752	downstream	0.003565
whole_cohort	LOAD	rs3094132	6	30219656	149323	downstream	0.00366
whole_cohort	LOAD	rs3132671	6	30210509	140176	downstream	0.003709
whole_cohort	LOAD	rs718254	6	30191261	120928	downstream	0.003748
whole_cohort	ADNI	rs2844697	6	30964531	894198	downstream	0.003768
whole_cohort	LOAD	rs1573295	6	30231289	160956	downstream	0.003871
whole_cohort	LOAD	rs2040486	6	30226325	155992	downstream	0.003871
whole_cohort	LOAD	rs3129690	6	30229077	158744	downstream	0.003871
whole_cohort	LOAD	rs3130391	6	30222862	152529	downstream	0.003871
whole_cohort	LOAD	rs1033569	6	29289994	776700	upstream	0.003932
whole_cohort	LOAD	rs3132667	6	30223809	153476	downstream	0.004013
whole_cohort	LOAD	rs765977	6	30189876	119543	downstream	0.004176
whole_cohort	LOAD	rs6457167	6	30187535	117202	downstream	0.004176
whole_cohort	LOAD	rs3130383	6	30211248	140915	downstream	0.004214
whole_cohort	CELL	rs2747453	6	29687165	379529	upstream	0.004271
whole_cohort	LOAD	rs9261539	6	30177305	106972	downstream	0.004317
whole_cohort	CELL	rs1633068	6	29756532	310162	upstream	0.004431
whole_cohort	CELL	rs6925061	6	30024508	42186	upstream	0.004503
whole_cohort	CELL	rs9261285	6	30068305	0	in gene	0.004503
whole_cohort	LOAD	rs7770592	6	29427969	638725	upstream	0.004543
whole_cohort	LOAD	rs969931	6	29527119	539575	upstream	0.004612
whole_cohort	CELL	rs3117294	6	29685551	381143	upstream	0.004657
whole_cohort	LOAD	rs954037	6	30235824	165491	downstream	0.004681
whole_cohort	LOAD	rs11752362	6	30293204	222871	downstream	0.004698
whole_cohort	ADNI	rs17875371	6	30492454	422121	downstream	0.004777
whole_cohort	LOAD	rs720831	6	29316740	749954	upstream	0.004826
whole_cohort	LOAD	rs4713208	6	29315801	750893	upstream	0.004941
whole_cohort	LOAD	rs9348821	6	29079425	987269	upstream	0.004996
whole_cohort	LOAD	rs28994870	6	30704979	634646	downstream	0.004999
whole_cohort	CELL	rs932338	6	29739155	327539	upstream	0.005109
whole_cohort	LOAD	rs6930435	6	29333444	733250	upstream	0.005233

	whole_cohort	LOAD	rs1014258	6	29342903	723791	upstream	0.005473
	whole_cohort	LOAD	rs9261543	6	30178872	108539	downstream	0.00555
	whole_cohort	CELL	rs1610628	6	29760431	306263	upstream	0.005553
	whole_cohort	CELL	rs9258866	6	29868746	197948	upstream	0.005813
	whole_cohort	CELL	rs3094170	6	29861487	205207	upstream	0.005926
	whole_cohort	LOAD	rs7758503	6	30300108	229775	downstream	0.005992
	whole_cohort	CELL	rs6923832	6	30094280	23947	downstream	0.006058
	whole_cohort	CELL	rs3129106	6	29113383	953311	upstream	0.006343
	whole_cohort	LOAD	rs2074464	6	29440750	625944	upstream	0.006817
	whole_cohort	LOAD	rs3094097	6	30666097	595764	downstream	0.006918
	whole_cohort	LOAD	rs3094093	6	30711850	641517	downstream	0.006957
	whole_cohort	CELL	rs9258525	6	29838563	228131	upstream	0.007511
	whole_cohort	CELL	rs2071653	6	29668150	398544	upstream	0.007646
	whole_cohort	LOAD	rs362529	6	29563090	503604	upstream	0.007786
	whole_cohort	LOAD	rs9257694	6	29306708	759986	upstream	0.007792
	whole_cohort	CELL	rs4713420	6	31025789	955456	downstream	0.007901
	whole_cohort	CELL	rs1737046	6	29767915	298779	upstream	0.007937
	whole_cohort	LOAD	rs29230	6	29608615	458079	upstream	0.008393
	whole_cohort	CELL	rs3132680	6	30105417	35084	downstream	0.008481
	whole_cohort	CELL	rs1611364	6	29711824	354870	upstream	0.008493
	whole_cohort	LOAD	rs2285801	6	30241715	171382	downstream	0.009176
	whole_cohort	LOAD	rs1362089	6	30242024	171691	downstream	0.009224
	whole_cohort	CELL	rs1264581	6	30329727	259394	downstream	0.009561
	whole_cohort	LOAD	rs3117073	6	29577047	489647	upstream	0.009754
**PTPRN2**								
	whole_cohort	LOAD	rs13221118	7	157007927	531129	downstream	0.0005736
	whole_cohort	LOAD	rs1263558	7	157670387	0	in gene	0.0008024
	whole_cohort	LOAD	rs1263546	7	157672674	0	in gene	0.001284
	whole_cohort	LOAD	rs6459771	7	157405571	133485	downstream	0.001764
	whole_cohort	LOAD	rs731305	7	157890147	0	in gene	0.001787
	whole_cohort	LOAD	rs1670340	7	158478329	0	in gene	0.002256
	whole_cohort	LOAD	rs10256092	7	158051694	0	in gene	0.002356
	whole_cohort	CELL	rs2602565	7	158833337	245541	upstream	0.002739
	whole_cohort	LOAD	rs2286842	7	157423398	115658	downstream	0.002979
	whole_cohort	LOAD	rs7799010	7	157074439	464617	downstream	0.003155
	whole_cohort	LOAD	rs4019377	7	157963122	0	in gene	0.003628
	whole_cohort	LOAD	rs1529835	7	157848976	0	in gene	0.003768
	whole_cohort	LOAD	rs7801805	7	157961789	0	in gene	0.004392
	whole_cohort	LOAD	rs2365999	7	157079100	459956	downstream	0.004516
	whole_cohort	CELL	rs1189202	7	158769102	181306	upstream	0.004666
	whole_cohort	LOAD	rs3793183	7	158928107	340311	upstream	0.005406
	whole_cohort	LOAD	rs1670339	7	158478446	0	in gene	0.005536
	whole_cohort	LOAD	rs10227782	7	157809603	0	in gene	0.005583
	whole_cohort	LOAD	rs10247026	7	158437902	0	in gene	0.005835
	whole_cohort	LOAD	rs6459830	7	158042035	0	in gene	0.006422
	whole_cohort	LOAD	rs4716739	7	157458159	80897	downstream	0.006554
	whole_cohort	LOAD	rs2301900	7	158918603	330807	upstream	0.006816
	whole_cohort	LOAD	rs12672574	7	158891460	303664	upstream	0.006877
	whole_cohort	LOAD	rs1242778	7	157768070	0	in gene	0.007093
	whole_cohort	CELL	rs7787974	7	158735478	147682	upstream	0.007309
	whole_cohort	LOAD	rs3815215	7	158911362	323566	upstream	0.007662
	whole_cohort	LOAD	rs7796348	7	157806382	0	in gene	0.007892
	whole_cohort	LOAD	rs7783724	7	158044664	0	in gene	0.008163

	whole_cohort	LOAD	rs4909239	7	158533394	0	in gene	0.008377
	whole_cohort	LOAD	rs6459743	7	157234413	304643	downstream	0.00851
	whole_cohort	LOAD	rs10081235	7	158530023	0	in gene	0.008597
	whole_cohort	LOAD	rs1263556	7	157683767	0	in gene	0.009073
	whole_cohort	CELL	rs842448	7	158773525	185729	upstream	0.009207
	whole_cohort	CELL	rs1307541	7	158782552	194756	upstream	0.009207
**RGS7**								
	whole_cohort	LOAD	rs316896	1	242166511	809281	upstream	0.000271
	whole_cohort	LOAD	rs11582266	1	242161096	803866	upstream	0.0005099
	whole_cohort	LOAD	rs598292	1	241641502	284272	upstream	0.0005458
	whole_cohort	ADNI	rs12030098	1	241584753	227523	upstream	0.00058
	whole_cohort	LOAD	rs1932440	1	241539788	182558	upstream	0.0006691
	whole_cohort	CELL	rs10802949	1	241341486	0	in gene	0.001056
	whole_cohort	LOAD	rs10803002	1	241927111	569881	upstream	0.001208
	whole_cohort	LOAD	rs12030098	1	241584753	227523	upstream	0.001899
	whole_cohort	ADNI	rs261858	1	240933455	0	in gene	0.002027
	whole_cohort	LOAD	rs1393299	1	242166488	809258	upstream	0.002081
	whole_cohort	LOAD	rs1550125	1	242134120	776890	upstream	0.002117
	whole_cohort	ADNI	rs12059322	1	241042132	0	in gene	0.002151
	whole_cohort	LOAD	rs1342445	1	240793548	0	in gene	0.002171
	whole_cohort	LOAD	rs1350224	1	240290590	484924	downstream	0.002195
	whole_cohort	LOAD	rs601675	1	241020311	0	in gene	0.002424
	whole_cohort	LOAD	rs16840310	1	240530862	244652	downstream	0.002428
	whole_cohort	LOAD	rs10926550	1	241723558	366328	upstream	0.002514
	whole_cohort	LOAD	rs6690415	1	242211692	854462	upstream	0.002604
	whole_cohort	LOAD	rs28593183	1	242032625	675395	upstream	0.002652
	whole_cohort	LOAD	rs12736879	1	240785720	0	in gene	0.002842
	whole_cohort	LOAD	rs922306	1	240010990	764524	downstream	0.003084
	whole_cohort	LOAD	rs10926538	1	241698746	341516	upstream	0.003324
	whole_cohort	ADNI	rs12753782	1	240724246	51268	downstream	0.004091
	whole_cohort	LOAD	rs10803013	1	242069776	712546	upstream	0.004171
	whole_cohort	LOAD	rs716527	1	240263854	511660	downstream	0.004202
	whole_cohort	ADNI	rs12088961	1	241049622	0	in gene	0.004299
	whole_cohort	LOAD	rs425246	1	242240957	883727	upstream	0.004551
	whole_cohort	LOAD	rs7513948	1	240658460	117054	downstream	0.004791
	whole_cohort	LOAD	rs316835	1	242185299	828069	upstream	0.004946
	whole_cohort	LOAD	rs6429223	1	240796772	0	in gene	0.00507
	whole_cohort	ADNI	rs9661049	1	240433950	341564	downstream	0.005357
	whole_cohort	LOAD	rs6696557	1	241127373	0	in gene	0.006169
	whole_cohort	LOAD	rs12140610	1	240054084	721430	downstream	0.006595
	whole_cohort	LOAD	rs12138459	1	241560314	203084	upstream	0.006637
	whole_cohort	ADNI	rs9662100	1	240415958	359556	downstream	0.007637
	whole_cohort	LOAD	rs12725593	1	241719362	362132	upstream	0.008054
	whole_cohort	LOAD	rs602676	1	241020592	0	in gene	0.008321
	whole_cohort	LOAD	rs11809579	1	242181092	823862	upstream	0.00845
	whole_cohort	LOAD	rs6690359	1	241727737	370507	upstream	0.008598
	whole_cohort	CELL	rs12026958	1	240871172	0	in gene	0.008638
	whole_cohort	ADNI	rs12138459	1	241560314	203084	upstream	0.008712
	whole_cohort	LOAD	rs10926178	1	240251749	523765	downstream	0.008799
	whole_cohort	ADNI	rs10495474	1	240712977	62537	downstream	0.008799
	whole_cohort	LOAD	rs1337088	1	239984202	791312	downstream	0.008869
	whole_cohort	LOAD	rs12096679	1	240448313	327201	downstream	0.009085
**SLC17A7**								

	whole_cohort	LOAD	rs8107595	19	49631462	189911	upstream	0.0002871
	whole_cohort	LOAD	rs1673041	19	50406131	964580	upstream	0.0009408
	whole_cohort	LOAD	rs643786	19	50352691	911140	upstream	0.001228
	whole_cohort	LOAD	rs281398	19	48673772	755626	downstream	0.001961
	whole_cohort	LOAD	rs11666105	19	48942559	486839	downstream	0.002194
	whole_cohort	LOAD	rs2270939	19	48939675	489723	downstream	0.002468
	whole_cohort	LOAD	rs648644	19	48707125	722273	downstream	0.002771
	whole_cohort	LOAD	rs4801774	19	48776354	653044	downstream	0.003055
	whole_cohort	CELL	rs11671777	19	48921667	507731	downstream	0.003355
	whole_cohort	LOAD	rs6509438	19	49576193	134642	upstream	0.003729
	whole_cohort	LOAD	rs8111317	19	48774884	654514	downstream	0.003964
	whole_cohort	LOAD	rs283526	19	49932604	491053	upstream	0.004599
	whole_cohort	LOAD	rs245110	19	50023638	582087	upstream	0.005055
	whole_cohort	LOAD	rs3745516	19	50423484	981933	upstream	0.005084
	whole_cohort	LOAD	rs13346402	19	49576992	135441	upstream	0.005107
	whole_cohort	LOAD	rs4002419	19	49050874	378524	downstream	0.005718
	whole_cohort	LOAD	rs1805419	19	48955846	473552	downstream	0.006047
	whole_cohort	LOAD	rs2681568	19	49489109	47558	upstream	0.006458
	whole_cohort	LOAD	rs8102979	19	49566636	125085	upstream	0.007797
	whole_cohort	LOAD	rs9304701	19	50047553	606002	upstream	0.008699
	whole_cohort	CELL	rs16982311	19	48838930	590468	downstream	0.009168
	whole_cohort	LOAD	rs1651543	19	50272484	830933	upstream	0.009746
**SLC25A11**								
	whole_cohort	LOAD	rs4790586	17	4184319	752811	downstream	0.0001099
	whole_cohort	LOAD	rs9907500	17	4104580	832550	downstream	0.0001491
	whole_cohort	LOAD	rs897022	17	4101239	835891	downstream	0.0001739
	whole_cohort	LOAD	rs8066945	17	4248872	688258	downstream	0.0002205
	whole_cohort	LOAD	rs11655342	17	4453079	484051	downstream	0.0002392
	whole_cohort	LOAD	rs882847	17	4479433	457697	downstream	0.0004754
	whole_cohort	LOAD	rs2325989	17	4296024	641106	downstream	0.0004786
	whole_cohort	LOAD	rs8078739	17	4345103	592027	downstream	0.0006328
	whole_cohort	LOAD	rs11078482	17	4125405	811725	downstream	0.0008814
	whole_cohort	LOAD	rs2603030	17	4029489	907641	downstream	0.0009178
	whole_cohort	ADNI	rs8072531	17	4848964	88166	downstream	0.001205
	whole_cohort	LOAD	rs1808820	17	4182538	754592	downstream	0.001267
	whole_cohort	LOAD	rs437649	17	5050428	110261	upstream	0.001908
	whole_cohort	CELL	rs7223183	17	4746489	190641	downstream	0.001953
	whole_cohort	CELL	rs9901248	17	5824379	884212	upstream	0.00204
	whole_cohort	ADNI	rs9893197	17	4854302	82828	downstream	0.00349
	whole_cohort	LOAD	rs11651767	17	4170103	767027	downstream	0.003988
	whole_cohort	ADNI	rs4790207	17	4599014	338116	downstream	0.00431
	whole_cohort	LOAD	rs17763879	17	4104396	832734	downstream	0.004477
	whole_cohort	ADNI	rs11871409	17	5825549	885382	upstream	0.004525
	whole_cohort	LOAD	rs3786046	17	5017064	76897	upstream	0.004721
	whole_cohort	ADNI	rs3816686	17	4551870	385260	downstream	0.00497
	whole_cohort	LOAD	rs1994979	17	4447694	489436	downstream	0.005174
	whole_cohort	LOAD	rs8077539	17	4846640	90490	downstream	0.005187
	whole_cohort	CELL	rs754814	17	4753738	183392	downstream	0.005349
	whole_cohort	CELL	rs11871409	17	5825549	885382	upstream	0.005447
	whole_cohort	LOAD	rs7210596	17	4161234	775896	downstream	0.005743
	whole_cohort	CELL	rs10491086	17	5745699	805532	upstream	0.006514
	whole_cohort	CELL	rs17175885	17	4013434	923696	downstream	0.006551
	whole_cohort	CELL	rs1109225	17	5818560	878393	upstream	0.006577

	whole_cohort	CELL	rs16955115	17	5795166	854999	upstream	0.006966
	whole_cohort	ADNI	rs4790654	17	4592386	344744	downstream	0.007072
	whole_cohort	CELL	rs952079	17	4416310	520820	downstream	0.007174
	whole_cohort	CELL	rs11078590	17	5825588	885421	upstream	0.007184
	whole_cohort	LOAD	rs2641269	17	5190603	250436	upstream	0.007712
	whole_cohort	CELL	rs9890937	17	4757212	179918	downstream	0.008056
	whole_cohort	ADNI	rs7218600	17	5825179	885012	upstream	0.008901
	whole_cohort	ADNI	rs3026106	17	5378804	438637	upstream	0.009232
	whole_cohort	ADNI	rs899446	17	4593366	343764	downstream	0.009414
	whole_cohort	CELL	rs3816686	17	4551870	385260	downstream	0.009777
**SNAP25**								
	whole_cohort	LOAD	rs6108803	20	11014318	706900	downstream	4.25E-07
	whole_cohort	LOAD	rs2206484	20	9783420	435274	upstream	0.0006811
	whole_cohort	LOAD	rs2232267	20	9516409	702285	upstream	0.0006907
	whole_cohort	LOAD	rs6086904	20	9486398	732296	upstream	0.0007806
	whole_cohort	LOAD	rs6056734	20	9596731	621963	upstream	0.0008713
	whole_cohort	LOAD	rs8118059	20	10814659	507241	downstream	0.001115
	whole_cohort	LOAD	rs6056656	20	9506385	712309	upstream	0.00113
	whole_cohort	LOAD	rs4813955	20	11027733	720315	downstream	0.001132
	whole_cohort	LOAD	rs2423511	20	10698446	391028	downstream	0.001241
	whole_cohort	LOAD	rs11087845	20	9424411	794283	upstream	0.00127
	whole_cohort	ADNI	rs362584	20	10273826	0	in gene	0.001454
	whole_cohort	LOAD	rs1028338	20	9383815	834879	upstream	0.001725
	whole_cohort	LOAD	rs2423456	20	9740093	478601	upstream	0.001775
	whole_cohort	CELL	rs2206484	20	9783420	435274	upstream	0.003033
	whole_cohort	LOAD	rs6032948	20	10778726	471308	downstream	0.003202
	whole_cohort	ADNI	rs1569832	20	10847651	540233	downstream	0.004113
	whole_cohort	LOAD	rs2327164	20	9318241	900453	upstream	0.004471
	whole_cohort	LOAD	rs6056740	20	9601228	617466	upstream	0.004507
	whole_cohort	LOAD	rs6108686	20	10730330	422912	downstream	0.004536
	whole_cohort	LOAD	rs7272444	20	9322459	896235	upstream	0.004556
	whole_cohort	LOAD	rs6056750	20	9610556	608138	upstream	0.004583
	whole_cohort	CELL	rs6056725	20	9593415	625279	upstream	0.004749
	whole_cohort	ADNI	rs2144184	20	10911149	603731	downstream	0.005336
	whole_cohort	LOAD	rs2276484	20	9470679	748015	upstream	0.005516
	whole_cohort	LOAD	rs6118591	20	9393872	824822	upstream	0.005785
	whole_cohort	ADNI	rs362569	20	10266084	0	in gene	0.00589
	whole_cohort	LOAD	rs2276483	20	9470384	748310	upstream	0.005974
	whole_cohort	LOAD	rs3025866	20	10255277	0	in gene	0.006337
	whole_cohort	LOAD	rs6039536	20	9653761	564933	upstream	0.006723
	whole_cohort	CELL	rs2210455	20	9938734	279960	upstream	0.006905
	whole_cohort	LOAD	rs6056871	20	9785003	433691	upstream	0.007574
	whole_cohort	CELL	rs6033042	20	11169788	862370	downstream	0.007679
	whole_cohort	LOAD	rs6056522	20	9311476	907218	upstream	0.008004
	whole_cohort	LOAD	rs1885563	20	9649589	569105	upstream	0.008044
	whole_cohort	LOAD	rs12233274	20	10699135	391717	downstream	0.008079
	whole_cohort	ADNI	rs6056505	20	9263134	955560	upstream	0.008119
	whole_cohort	ADNI	rs362562	20	10262537	0	in gene	0.00864
	whole_cohort	CELL	rs6039837	20	10321808	14390	downstream	0.00909
	whole_cohort	CELL	rs2327225	20	9767479	451215	upstream	0.009303
	whole_cohort	CELL	rs7272324	20	10321684	14266	downstream	0.009372
	whole_cohort	LOAD	rs2206476	20	9689595	529099	upstream	0.009728
	whole_cohort	LOAD	rs1569625	20	9759792	458902	upstream	0.009754

	whole_cohort	LOAD	rs362584		20	10273826	0	in gene	0.009924
**SYP**									
	whole_cohort	LOAD	rs6520497	X		49978553	778351	upstream	8.85E-06
	whole_cohort	LOAD	rs7059734	X		50104046	903844	upstream	2.31E-05
	whole_cohort	LOAD	rs3027514	X		48917662	270150	downstream	2.38E-05
	whole_cohort	LOAD	rs6651676	X		49123318	64494	downstream	6.29E-05
	whole_cohort	LOAD	rs6651677	X		49123439	64373	downstream	6.57E-05
	whole_cohort	LOAD	rs7886979	X		50054556	854354	upstream	0.000216
	whole_cohort	LOAD	rs5905692	X		48483366	704446	downstream	0.0003941
	whole_cohort	LOAD	rs7888633	X		50158444	958242	upstream	0.0006029
	whole_cohort	LOAD	rs17332716	X		50199585	999383	upstream	0.0009899
	whole_cohort	LOAD	rs6520321	X		48214972	972840	downstream	0.002156
	whole_cohort	LOAD	rs17174048	X		49983244	783042	upstream	0.002358
	whole_cohort	LOAD	rs5906595	X		48235710	952102	downstream	0.002377
	whole_cohort	LOAD	rs6610040	X		49743822	543620	upstream	0.002412
	whole_cohort	LOAD	rs5906593	X		48235498	952314	downstream	0.002582
	whole_cohort	LOAD	rs17148347	X		49281452	81250	upstream	0.008353
**TPI1**									
	whole_cohort	LOAD	rs4376959		12	6632649	234771	upstream	2.49E-05
	whole_cohort	LOAD	rs1045553		12	6638128	229292	upstream	2.86E-05
	whole_cohort	LOAD	rs10744062		12	7161163	290217	downstream	0.0001165
	whole_cohort	LOAD	rs7306351		12	7835164	964218	downstream	0.0002164
	whole_cohort	LOAD	rs744167		12	6944744	73798	downstream	0.0003445
	whole_cohort	LOAD	rs10743732		12	7324701	453755	downstream	0.001458
	whole_cohort	LOAD	rs1990385		12	6285468	581952	upstream	0.001486
	whole_cohort	LOAD	rs7489044		12	5900428	966992	upstream	0.001794
	whole_cohort	ADNI	rs5446		12	6847297	20123	upstream	0.00198
	whole_cohort	LOAD	rs17727473		12	7243137	372191	downstream	0.002713
	whole_cohort	LOAD	rs2091781		12	6763345	104075	upstream	0.00285
	whole_cohort	LOAD	rs6489726		12	6632820	234600	upstream	0.003083
	whole_cohort	LOAD	rs12305746		12	6161316	706104	upstream	0.003159
	whole_cohort	LOAD	rs4883263		12	7496887	625941	downstream	0.003173
	whole_cohort	ADNI	rs6488567		12	7669095	798149	downstream	0.00341
	whole_cohort	LOAD	rs11054072		12	7471127	600181	downstream	0.003723
	whole_cohort	LOAD	rs4149579		12	6338190	529230	upstream	0.003781
	whole_cohort	LOAD	rs3741920		12	6829707	37713	upstream	0.003859
	whole_cohort	ADNI	rs4149577		12	6338355	529065	upstream	0.004125
	whole_cohort	LOAD	rs4883422		12	7145730	274784	downstream	0.00447
	whole_cohort	LOAD	rs11064410		12	6810402	57018	upstream	0.004757
	whole_cohort	LOAD	rs3782711		12	6074014	793406	upstream	0.004896
	whole_cohort	LOAD	rs4883211		12	7289036	418090	downstream	0.004991
	whole_cohort	LOAD	rs7962629		12	7059465	188519	downstream	0.005131
	whole_cohort	LOAD	rs797770		12	6193990	673430	upstream	0.005228
	whole_cohort	ADNI	rs12310569		12	6588186	279234	upstream	0.005997
	whole_cohort	CELL	rs6488388		12	7510864	639918	downstream	0.007272
	whole_cohort	LOAD	rs12810426		12	6034971	832449	upstream	0.007296
	whole_cohort	LOAD	rs7970379		12	7299191	428245	downstream	0.007555
	whole_cohort	LOAD	rs7311672		12	7057809	186863	downstream	0.008039
	whole_cohort	LOAD	rs6488338		12	7479546	608600	downstream	0.008339
	whole_cohort	ADNI	rs2238114		12	6856575	10845	upstream	0.008561
	whole_cohort	ADNI	rs4149576		12	6339948	527472	upstream	0.00864
	whole_cohort	LOAD	rs2238114		12	6856575	10845	upstream	0.008859
	whole_cohort	LOAD	rs11064498		12	7064202	193256	downstream	0.009973

	whole_cohort	LOAD	rs10772425	12	7500716	629770	downstream	0.009989
**UQCRC1**								
	whole_cohort	LOAD	rs12107252	3	48653882	44217	upstream	0.001862
	whole_cohort	LOAD	rs12107418	3	48652353	42688	upstream	0.001862
	whole_cohort	LOAD	rs2276852	3	48629489	19824	upstream	0.002589
	whole_cohort	LOAD	rs1471217	3	48343404	255595	downstream	0.00261
	whole_cohort	LOAD	rs7645082	3	47960895	638104	downstream	0.003588
	whole_cohort	LOAD	rs1464615	3	47612211	986788	downstream	0.006552
**YWHAB**								
	whole_cohort	LOAD	rs4812866	20	44796824	88775	upstream	3.56E-05
	whole_cohort	LOAD	rs6073519	20	44689615	195984	upstream	0.000135
	whole_cohort	LOAD	rs6031807	20	44807795	77804	upstream	0.0001859
	whole_cohort	LOAD	rs4812848	20	44680636	204963	upstream	0.0003483
	whole_cohort	LOAD	rs6031752	20	44705832	179767	upstream	0.0005413
	whole_cohort	LOAD	rs11905122	20	45339965	431433	downstream	0.0005857
	whole_cohort	LOAD	rs4812851	20	44691642	193957	upstream	0.000748
	whole_cohort	LOAD	rs11906229	20	44779788	105811	upstream	0.0008029
	whole_cohort	LOAD	rs3827040	20	45886919	978387	downstream	0.001353
	whole_cohort	LOAD	rs9346	20	44197709	687890	upstream	0.001977
	whole_cohort	LOAD	rs6094091	20	45060232	151700	downstream	0.003094
	whole_cohort	LOAD	rs6876	20	44906459	0	in gene	0.003633
	whole_cohort	LOAD	rs6103674	20	44198069	687530	upstream	0.004022
	whole_cohort	LOAD	rs197666	20	45842586	934054	downstream	0.004885
	whole_cohort	LOAD	rs6017235	20	43991951	893648	upstream	0.005301
	whole_cohort	LOAD	rs4812867	20	44796881	88718	upstream	0.005355
	whole_cohort	ADNI	rs736824	20	44406019	479580	upstream	0.007246
	whole_cohort	LOAD	rs7348267	20	45455745	547213	downstream	0.007711
	whole_cohort	LOAD	rs6031256	20	43954976	930623	upstream	0.007881
	whole_cohort	LOAD	rs6032205	20	45454158	545626	downstream	0.007983
	whole_cohort	ADNI	rs3092796	20	44533829	351770	upstream	0.009421

Supplementary Table 7b

SNPs present in various cohorts using thresholds of 200 kb distance, p < 5 x 10-4.

Gene	*Cohort*	*Study*	*SNP*	*Chromo-some*	*SNP location*	*Distance from gene*	*Relative to gene*	*GWAS p value*
**ATP1A3**								
	female_apoe4+	LOAD	rs717225	19	41889214	77262	downstream	2.46E-05
	female_apoe4+	LOAD	rs7260129	19	41922092	44384	downstream	6.15E-05
	female_apoe4+	LOAD	rs12104228	19	41894376	72100	downstream	0.0001456
	apoe4+	LOAD	rs717225	19	41889214	77262	downstream	9.35E-05
	apoe4+	LOAD	rs7260129	19	41922092	44384	downstream	0.0004602
	apoe4+	LOAD	rs12104228	19	41894376	72100	downstream	0.0003404
	female	LOAD	rs717225	19	41889214	77262	downstream	4.34E-05
	female	LOAD	rs7260129	19	41922092	44384	downstream	1.92E-05
	female	LOAD	rs12104228	19	41894376	72100	downstream	0.0001707
	whole_cohort	LOAD	rs717225	19	41889214	77262	downstream	3.43E-05
	whole_cohort	LOAD	rs7260129	19	41922092	44384	downstream	3.23E-05
	whole_cohort	LOAD	rs12104228	19	41894376	72100	downstream	0.0001055
**ATP6V1E1**								
	male_apoe4+	CELL	rs8141657	22	17509361	82775	downstream	0.0003088
	whole_cohort	LOAD	rs174294	22	17515555	76581	downstream	0.0001572
	whole_cohort	LOAD	rs1296792	22	17538826	53310	downstream	6.04E-05
	whole_cohort	LOAD	rs1296793	22	17539250	52886	downstream	6.04E-05
	whole_cohort	LOAD	rs1296794	22	17541914	50222	downstream	5.18E-05
**ATP6V1G2**								
	female_apoe4-	ADNI	rs2848713	6	31416701	127750	downstream	0.0001303
	female_apoe4-	ADNI	rs6933050	6	31375854	168597	downstream	0.0003496
	female_apoe4-	ADNI	rs7770216	6	31372833	171618	downstream	0.000105
	female_apoe4-	ADNI	rs9266327	6	31362841	181610	downstream	0.0001393
	female_apoe4-	ADNI	rs9266395	6	31367788	176663	downstream	0.0001876
	female_apoe4-	ADNI	rs9266399	6	31368028	176423	downstream	9.72E-05
	female_apoe4-	ADNI	rs9266409	6	31368790	175661	downstream	9.72E-05
	apoe4+	CELL	rs2523619	6	31350366	194085	downstream	0.000103
	female	LOAD	rs17201046	6	31731795	184947	upstream	0.0001102
**BNIP3**								
	apoe4-	LOAD	rs11156481	10	131815217	152483	downstream	0.0004396
**C14orf2**								
	female_apoe4-	LOAD	rs1744296	14	104101384	179818	upstream	0.0003485
	female_apoe4-	LOAD	rs12891477	14	103866421	45867	downstream	0.0003656
**C14orf132**								
	whole_cohort	LOAD	rs10498647	14	95915004	124320	upstream	7.07E-05
**CACNG3**								
	female_apoe4+	CELL	rs890846	16	24495834	133418	downstream	1.48E-05
	apoe4+	CELL	rs890846	16	24495834	133418	downstream	0.0002233
	whole_cohort	LOAD	rs7199880	16	24413065	50649	downstream	0.0001467
**GNG3**								
	whole_cohort	LOAD	rs2730034	11	62809760	100554	downstream	0.000427
**GOT2**								
	female_apoe4-	CELL	rs1437176	16	58922575	188218	upstream	5.73E-05
	female_apoe4+	LOAD	rs6499977	16	58773637	39280	upstream	0.0003028
	apoe4+	LOAD	rs6499977	16	58773637	39280	upstream	3.38E-05

apoe4+	LOAD	rs7201899	16	58677565	29566	downstream	0.0001738
**female**	**LOAD**	**rs6499977**	**16**	**58773637**	**39280**	**upstream**	**5.84E-05**
**female**	**LOAD**	**rs7201899**	**16**	**58677565**	**29566**	**downstream**	**0.0002543**
**female**	**CELL**	**rs1437176**	**16**	**58922575**	**188218**	**upstream**	**0.0003811**
**whole_cohort**	**LOAD**	**rs6499969**	**16**	**58700302**	**6829**	**downstream**	**0.0001352**
**whole_cohort**	**LOAD**	**rs6499977**	**16**	**58773637**	**39280**	**upstream**	**3.11E-06**
**whole_cohort**	**LOAD**	**rs7201899**	**16**	**58677565**	**29566**	**downstream**	**1.74E-05**
**whole_cohort**	**CELL**	**rs1437176**	**16**	**58922575**	**188218**	**upstream**	**0.0004375**

MAGED1

female	LOAD	rs14115	X	51923689	21335	downstream	0.0001281
whole_cohort	LOAD	rs14115	X	51923689	21335	downstream	0.0001216

MRPS11

**female_apoe4+**	**LOAD**	**rs7172522**	**15**	**88636787**	**158157**	**downstream**	**0.000109**
**female_apoe4+**	**LOAD**	**rs11638243**	**15**	**88636444**	**157814**	**downstream**	**0.0001493**
**female_apoe4+**	**CELL**	**rs1530310**	**15**	**88279695**	**187758**	**upstream**	**0.0004824**
apoe4+	LOAD	rs1442291	15	88381490	85963	upstream	0.000459
apoe4+	LOAD	rs3743473	15	88630816	152186	downstream	0.0001077
apoe4+	LOAD	rs6496484	15	88442442	25011	upstream	6.49E-05
apoe4+	LOAD	rs7172522	15	88636787	158157	downstream	0.0004983
apoe4+	LOAD	rs8027765	15	88626626	147996	downstream	0.0002096
female	LOAD	rs3743473	15	88630816	152186	downstream	0.0002776
female	LOAD	rs6496484	15	88442442	25011	upstream	0.0002389
female	LOAD	rs7172522	15	88636787	158157	downstream	1.82E-05
female	LOAD	rs8027765	15	88626626	147996	downstream	0.0002005
female	LOAD	rs11638243	15	88636444	157814	downstream	3.81E-05
whole_cohort	LOAD	rs3743473	15	88630816	152186	downstream	4.37E-06
whole_cohort	LOAD	rs6496484	15	88442442	25011	upstream	5.88E-06
whole_cohort	LOAD	rs7172522	15	88636787	158157	downstream	0.0001045
whole_cohort	LOAD	rs7176734	15	88472054	0	in gene	0.0001258
whole_cohort	LOAD	rs8027765	15	88626626	147996	downstream	7.34E-06
whole_cohort	LOAD	rs9920906	15	88471117	0	in gene	0.0003131
whole_cohort	LOAD	rs11638243	15	88636444	157814	downstream	0.0003849

NEUROD6

**female_apoe4+**	**LOAD**	**rs6972352**	**7**	**31495240**	**154316**	**upstream**	**0.0004897**
**female_apoe4+**	**ADNI**	**rs1917011**	**7**	**31274254**	**63207**	**downstream**	**3.82E-05**
**female_apoe4+**	**ADNI**	**rs2159766**	**7**	**31250957**	**86504**	**downstream**	**3.82E-05**
**female_apoe4+**	**ADNI**	**rs12701070**	**7**	**31250840**	**86621**	**downstream**	**3.82E-05**
apoe4+	ADNI	rs7801993	7	31359718	18794	upstream	0.000318
apoe4+	ADNI	rs11983505	7	31426128	85204	upstream	8.96E-05
apoe4+	ADNI	rs13231468	7	31367826	26902	upstream	0.0001188
female	LOAD	rs6972352	7	31495240	154316	upstream	4.45E-05
whole_cohort	LOAD	rs6972352	7	31495240	154316	upstream	0.0002405

PTPRN2

female_apoe4+	CELL	rs12540718	7	158030283	0	in gene	0.0001116
female	LOAD	rs4019377	7	157963122	0	in gene	0.000301
male_apoe4-	CELL	rs842448	7	158773525	185729	upstream	0.0002957
male_apoe4-	CELL	rs959489	7	158760599	172803	upstream	0.000498
male_apoe4-	CELL	rs1307541	7	158782552	194756	upstream	0.0002957
male	CELL	rs842448	7	158773525	185729	upstream	0.0004751
male	CELL	rs1307541	7	158782552	194756	upstream	0.0004751

RGS7

female_apoe4+	LOAD	rs796786	1	240839919	0	in gene	4.84E-05
apoe4-	LOAD	rs2994968	1	241440253	83023	upstream	0.0002546
apoe4+	CELL	rs2815861	1	241227435	0	in gene	0.0002952
apoe4+	CELL	rs6691111	1	240639869	135645	downstream	6.98E-05
male	ADNI	rs261804	1	240904170	0	in gene	0.0004353

SLC17A7

whole_cohort	LOAD	rs8107595	19	49631462	189911	upstream	0.0002871

SLC25A11

apoe4-	LOAD	rs8072531	17	4848964	88166	downstream	0.00037

SNAP25

**male_apoe4+**	**LOAD**	**rs6077693**	**20**	**10226268**	**0**	**in gene**	**0.0002909**
**male_apoe4+**	**CELL**	**rs6032806**	**20**	**10142775**	**75919**	**upstream**	**0.000429**

SYP

female	LOAD	rs6651676	X	49123318	64494	downstream	0.0001066
female	LOAD	rs6651677	X	49123439	64373	downstream	0.0001109
male_apoe4-	CELL	rs3027471	X	49033820	153992	downstream	0.0004819
whole_cohort	LOAD	rs6651676	X	49123318	64494	downstream	6.29E-05
whole_cohort	LOAD	rs6651677	X	49123439	64373	downstream	6.57E-05

TPI1

female_apoe4+	LOAD	rs744167	12	6944744	73798	downstream	1.96E-05
apoe4+	LOAD	rs744167	12	6944744	73798	downstream	5.13E-05
whole_cohort	LOAD	rs744167	12	6944744	73798	downstream	0.0003445

YWHAB

female_apoe4-	LOAD	rs2903760	20	44827103	58496	upstream	0.000472
apoe4+	LOAD	rs4812851	20	44691642	193957	upstream	0.0004058
apoe4+	LOAD	rs6031807	20	44807795	77804	upstream	0.0001985
apoe4+	LOAD	rs6073519	20	44689615	195984	upstream	7.74E-05
male_apoe4+	LOAD	rs4812851	20	44691642	193957	upstream	0.0001311
male_apoe4+	LOAD	rs6073519	20	44689615	195984	upstream	8.09E-05
male	LOAD	rs6073519	20	44689615	195984	upstream	0.000335
whole_cohort	LOAD	rs4812866	20	44796824	88775	upstream	3.56E-05
whole_cohort	LOAD	rs6031807	20	44807795	77804	upstream	0.0001859
whole_cohort	LOAD	rs6073519	20	44689615	195984	upstream	0.000135

Supplementary Table 8. Genes modulated in a consistent direction in disease in multiple gene expression datasets.

Genes downregulated in disease in multiple gene expression datasets

Down in disease

Datasets

GSE1297
GSE15222
GSE36980
GSE44772

SCG3	RGS7	NME1
TPI1	GOT2	MAGED1
MRPS11	ENC1	SLC17A7
PGAM1	GNG3	ATP6V1E1
ATP5B	PTPRN2	SYP
SLC25A11	PPP1R11	ATP1A3
KPNA2	YWHAB	ATP6V1G2
NEDD8	BNIP3	C14orf2
AP3B2	SNAP25	CACNG3
C14orf132	NEUROD6	UQCRC1

GSE1297
GSE15222
GSE36980
GSE5281

MRPS11	GOT2	BNIP3	SYP
ATP5B	ATP6V1G2	SNAP25	ATP1A3
SLC25A11	PTPRN2	NEUROD6	GNG3
AP3B2	PPP1R11	MAGED1	C14orf2
C14orf132	YWHAB	SLC17A7	CACNG3
RGS7	CFL1	ATP6V1E1	UQCRC1
TPI1			

GSE1297
GSE15222
GSE44772
GSE5281

ASNA1	SLC25A11	NDUFA10	GPI	SHANK2	SLC35B1
LMO4	GABRA2	LDOC1	TTC3	CDK5	SYP
YWHAB	ATP5C1	GOT2	MPV17	GPHN	FKBP1B
ATP6V1H	APEX1	ATP1A3	TUBG2	SNAP25	GNG3
TPI1	AP3B2	SRD5A1	IDS	ATP6V1G2	SST
C14orf132	ARHGEF9	GOT1	PPP1R11	NEUROD6	C14orf2
MRPS11	ARF3	LDHA	NMNAT2	MAGED1	PPP3CA
ATP5B	BNIP3	LZTS1	PEX11B	SLC17A7	CACNG3
GABBR1	RAB11A	PTPRN2	NGFRAP1	ATP6V1E1	UQCRC1
GLS	PFKM	YWHAZ	CADPS	CHN1	
NDRG4	RGS7	DGKZ	UCHL1	AK5	

GSE15222
GSE36980
GSE44772
GSE5281

FXYD7	DDX41	SLC25A11	C12orf10	SV2B	C14orf132
ATP5J2	MAGED1	WSB2	FABP3	NEUROD6	SDHB
LRFN3	TRAPPC5	CRMP1	ATP5B	SYNGR1	RGS7
TPI1	SYT5	PTPRN2	DCTN1	SYNGR3	YWHAB
HPCA	SYP	DPYSL4	GABRA1	SLC17A7	ATP5G1
SEMA4F	GNG3	GFRA2	PGBD5	ATP6V1E1	ATP6V1G2
MICAL2	POP4	POLR2E	STMN2	ATP1A3	ANXA6
NAPA	ACLY	RAP1GDS1	AP3D1	C14orf2	CHRM1
GOT2	UQCRC1	BNIP3	MAPK9	PSMD8	PPP2R2C
ATP6V0D1	ELAVL4	PPP1R11	RAB15	EEF1A2	STS
ZFP64	COPS8	MKKS	ATP6V0C	BCL11A	SRM
MRPL37	ATP6V1F	NPTXR	MRPS18A	MRPS11	BSCL2
HK1	GBA	GAS7	PPEF1	PIN1	BAI2
KCNF1	COPS4	EHD3	TOMM70A	AP3B2	VAMP2
SLC25A4	LARGE	LRPAP1	SERPINF1	IDH3G	FIBP

SNAP25	NPTX2	VAPB	RAD51C	NEFL	NEDD4L
PLD3	RUVBL2	STXBP1	THY1	TLN2	KIAA1045
CACNG3	PTPRN				

	EPHA4	CAP2	ST6GALNAC5	MET
GSE1297	TPI1	RGS7	BNIP3	SYP
GSE36980	C14orf132	GOT2	SNAP25	ATP1A3
GSE44772	MRPS11	GNG3	NEUROD6	ATP6V1G2
GSE5281	ATP5B	PTPRN2	MAGED1	C14orf2
	SLC25A11	PPP1R11	SLC17A7	CACNG3
	AP3B2	YWHAB	ATP6V1E1	UQCRC1

Genes upregulated in disease in multiple gene expression datasets

Up in datasets:

GSE1297
GSE15222
GSE36980
GSE44772

-

GSE1297
GSE15222
GSE36980
GSE5281

-

GSE1297
GSE15222
GSE44772
GSE5281

MAP4K4	KTN1	CASC3
EHD1	ZHX3	FYCO1
JARID2	NPAS3	SASH1
PCSK5	SLC12A7	VAT1
HIPK2	NAV2	TBL1X
ANP32B	PTBP1	PTMA
EIF5B	TFEB	MXI1
MAPKAPK2	RXRA	NOTCH1
ABCA1	CTBP2	RBBP6

GSE15222
GSE36980
GSE44772
GSE5281

ARHGEF10
COLEC12
H3F3A

GSE1297
GSE36980
GSE44772
GSE5281

-

Supplementary Table 9 Affected vs Control comparison, stratified by gender

Gene	Probe	Dataset	M			F		
			FC	nominal p	FDR p	FC	nominal p	FDR p
**NEUROD6**	10025910121	GSE44772	-1.2336491	**2.26E-08**	**7.11E-06**	-1.1299804	**0.00059288**	*0.06064708*
	220045_at	GSE1297	-1.4420197	**0.02970883**	0.51157101	-2.1051808	**0.00106055**	**0.02198478**
	220045_at	GSE5281	-2.0415758	**0.00022875**	**0.00521989**	-1.6017269	**0.02100019**	0.16183694
	8138882	GSE36980	-2.3734997	**0.01492839**	0.36967773	-2.279541	**0.00394971**	0.1770335
	GI_27475984-S	GSE15222	-2.1569755	**6.35E-15**	**9.16E-12**	-1.9112995	**3.97E-12**	**1.91E-10**
**SNAP25**	10025909548	GSE44772	-1.1089471	**0.00030481**	**0.00150566**	-1.0490635	0.2065026	0.52889959
	10023826155	GSE44772	-1.135418	**0.00068959**	**0.00287672**	-1.0586663	0.2385865	0.56168282
	202507_s_at	GSE1297	-2.7274539	**0.03477811**	0.52601722	-9.8566541	**8.61E-05**	**0.00867944**
	202507_s_at	GSE5281	-2.5519022	**0.02984051**	0.12499269	-3.9177111	**0.00923765**	0.12308374
	202508_s_at	GSE1297	-3.1330418	**0.01202105**	0.44444713	-2.6981031	**0.01603544**	*0.08427302*
	202508_s_at	GSE5281	-1.309359	0.28658203	0.51071945	-1.8040611	*0.07553897*	0.25750317
	1556629_a_at	GSE1297	-			-		
	1556629_a_at	GSE5281	-2.6860378	**0.00016496**	**0.00418716**	-1.5471424	0.15235314	0.35194811
	8060963	GSE36980	-1.2592732	**0.0184594**	0.37407034	-1.8339166	**0.00123207**	0.15480796
	GI_18765734-A	GSE15222	-1.3256363	**0.03946573**	*0.07497882*	-2.3431188	**2.61E-08**	**2.17E-07**

(Largest 2 datasets by sample size denoted by underlining)
Each probe available on each platform for these genes was included.

Supplementary Table 10a. Genes targeted by NEUROD6-relevant compounds of interest, based on literature and data mining.

Drug	Target(s)	Source
sodium phenylbutyrate	HDAC Inhibitor	http://www.ncbi.nlm.nih.gov/pubmed/21902286
arachidonic acid	Chloride channel protein 2, Prostaglandin G/H synthase 1	http://bidd.nus.edu.sg/group/TTD/ZFTTDDRUG.asp?ID=DNC000249, http://bidd.nus.edu.sg/group/TTD/ZFTTDDRUG.asp?ID=DNC002453
2-deoxy-D-glucose		
fasudil	Rho-associated protein kinase 1	http://bidd.nus.edu.sg/group/TTD/ZFTTDDRUG.asp?ID=DNC000640
nordihydroguaiaretic acid	Acetyl-CoA acetyltransferase, mitochondrial, Arachidonate 5-lipoxygenase	http://bidd.nus.edu.sg/group/TTD/ZFTTDDRUG.asp?ID=DNC001037
monastrol	Kinesin-like protein KIF11	http://bidd.nus.edu.sg/group/TTD/ZFTTDDRUG.asp?ID=DNC003165
tacrolimus	Calcineurin	http://bidd.nus.edu.sg/group/TTD/ZFTTDDRUG.asp?ID=DAP000162
quercetin	Lyso-PAF: acetyl-CoA acetyltransferase, Phosphatidylinositol-4,5-bisphosphate 3-kinase catalytic subunit gamma isoform	http://bidd.nus.edu.sg/group/TTD/ZFTTDDRUG.asp?ID=DAP001419, http://bidd.nus.edu.sg/group/TTD/ZFTTDDRUG.asp?ID=DNC003453
sulindac	Aldose reductase	http://bidd.nus.edu.sg/group/TTD/ZFTTDDRUG.asp?ID=DAP000569
troglitazone	Peroxisome proliferator activated receptor gamma	http://bidd.nus.edu.sg/group/TTD/ZFTTDDRUG.asp?ID=DAP001337
staurosporine	Inhibitor of nuclear factor kappa B kinase beta subunit, Protein kinase C, Protein kinase D, 3-phosphoinositide-dependent protein kinase 1, Cell division protein kinase 2, Glycogen synthase kinase-3 beta, Phosphatidylinositol-4,5-bisphosphate 3-kinase catalytic subunit gamma isoform, Protein kinase C theta type, Proto-oncogene tyrosine-protein kinase LCK, Tyrosine-protein kinase ITK/TSK, Tyrosine-protein kinase SYK	http://bidd.nus.edu.sg/group/TTD/ZFTTDDRUG.asp?ID=DNC001380, http://bidd.nus.edu.sg/group/TTD/ZFTTDDRUG.asp?ID=DNC002412, http://bidd.nus.edu.sg/group/TTD/ZFTTDDRUG.asp?ID=DNC002782
troglitazone	Peroxisome proliferator activated receptor gamma	http://xin.cz3.nus.edu.sg/group/TTD/ZFTTDDRUG.asp?ID=DAP001337
thalidomide	Tumor necrosis factor receptor	http://xin.cz3.nus.edu.sg/group/TTD/ZFTTDDRUG.asp?ID=DAP000865
CP-944629		
mercaptopurine	DNA, Inosine-5'-monophosphate dehydrogenase	http://xin.cz3.nus.edu.sg/group/TTD/ZFTTDDRUG.asp?ID=DAP000147
haloperidol	D(2) dopamine receptor	http://xin.cz3.nus.edu.sg/group/TTD/ZFTTDDRUG.asp?ID=DAP000313
exisulind		
sirolimus	Serine/threonine-protein kinase mTOR	http://xin.cz3.nus.edu.sg/group/TTD/ZFTTDDRUG.asp?ID=DAP000663
tanespimycin	Heat shock protein HSP 90	http://xin.cz3.nus.edu.sg/group/TTD/ZFTTDDRUG.asp?ID=DCL000242
suramin sodium	Follicle-stimulating hormone receptor	http://xin.cz3.nus.edu.sg/group/TTD/ZFTTDDRUG.asp?ID=DAP001031
genistein	DNA topoisomerase 2-alpha, Estrogen receptor, Estrogen receptor beta, Peroxisome proliferator activated receptor gamma	http://xin.cz3.nus.edu.sg/group/TTD/ZFTTDDRUG.asp?ID=DNC002738, http://xin.cz3.nus.edu.sg/group/TTD/ZFTTDDRUG.asp?ID=DCL000330
erastin	Voltage-dependent anion-selective channel protein 2	https://www.bindingdb.org/bind/chemsearch/marvin/MolStructure.jsp?monomerid=50376126

clofibrate	Lipoprotein lipase	http://xin.cz3.nus.edu.sg/group/TTD/ZFTTDDRUG.asp?ID=DAP000262
LY-294002	DNA-dependent protein kinase, FK506 binding protein 12, Glycogen synthase kinase-3 beta, MAP kinase ERK2, MAP kinase p38 alpha, MAP kinase p38 beta, MAP kinase p38 gamma, PI3-kinase p110-beta subunit, PI3-kinase p110-delta subunit, PI3-kinase p110-gamma subunit, Protein kinase C alpha, Rho-associated protein kinase 2, Ribosomal protein S6 kinase alpha 5, Serine/threonine-protein kinase AKT, Serine/threonine-protein kinase Chk1, Serine/threonine-protein kinase PLK1, Serine/threonine-protein kinase Sgk1, Tyrosine-protein kinase LCK, c-Jun N-terminal kinase 1	http://xin.cz3.nus.edu.sg/group/TTD/ZFTTDDRUG.asp?ID=DNC003512
tanespimycin	Heat shock protein HSP 90	http://xin.cz3.nus.edu.sg/group/TTD/ZFTTDDRUG.asp?ID=DCL000242
prednisolone	Corticosteroid-binding globulin	http://xin.cz3.nus.edu.sg/group/TTD/ZFTTDDRUG.asp?ID=DAP000419
fulvestrant	Estrogen receptor	http://xin.cz3.nus.edu.sg/group/TTD/ZFTTDDRUG.asp?ID=DAP000319
meteneprost		
monorden		
tretinoin	Retinoic acid receptor	http://xin.cz3.nus.edu.sg/group/TTD/ZFTTDDRUG.asp?ID=DAP001221
nifedipine	Voltage-dependent calcium channel subunit alpha-2/delta-1	http://xin.cz3.nus.edu.sg/group/TTD/ZFTTDDRUG.asp?ID=DAP000529
sulindac sulfide	Cyclooxygenase	http://xin.cz3.nus.edu.sg/group/TTD/ZFTTDDRUG.asp?ID=DAP001429
wortmannin	Phosphatidylinositol 3-kinase, Phosphatidylinositol-4,5-bisphosphate 3-kinase catalytic subunit, delta isoform	http://xin.cz3.nus.edu.sg/group/TTD/ZFTTDDRUG.asp?ID=DNC001514
MK-886	5-lipoxygenase activating protein, Arachidonate 5-lipoxygenase, Prostaglandin G/H synthase 2	http://xin.cz3.nus.edu.sg/group/TTD/ZFTTDDRUG.asp?ID=DNC000953
PF-01378883-00		
iloprost	Prostaglandin E2 receptor, EP2 subtype	http://xin.cz3.nus.edu.sg/group/TTD/ZFTTDDRUG.asp?ID=DAP000273

Supplementary Table 10b. Genes targeted by SNAP25-relevant compounds of interest, based on literature and data mining.

Drug	Target(s)	Source
valproic acid	Histone deacetylase	http://bidd.nus.edu.sg/group/TTD/ZFTTDDRUG.asp?ID=DAP000831
guanabenz	Alpha-2 adrenergic receptor	http://bidd.nus.edu.sg/group/TTD/ZFTTDDRUG.asp?ID=DAP000232
karakoline	Neuronal acetylcholine receptor (alpha7 nAChR)	http://www.bindingdb.org/bind/chemsearch/marvin/MolStructure.jsp?monomerid=50454431
tetracycline	30S ribosomal subunit	http://bidd.nus.edu.sg/group/TTD/ZFTTDDRUG.asp?ID=DAP000032
diloxanide		
metoprolol	Beta-1 adrenergic receptor	http://bidd.nus.edu.sg/group/TTD/ZFTTDDRUG.asp?ID=DAP000481
yohimbic acid	Alpha-2 adrenergic receptor	http://bidd.nus.edu.sg/group/TTD/ZFTTDDRUG.asp?ID=DAP000087
azapropazone		
proguanil	Dihydrofolate reductase	http://bidd.nus.edu.sg/group/TTD/ZFTTDDRUG.asp?ID=DAP000634

Supplementary Table 11 Female vs Male comparison, stratified by disease status

Gene	Probe	Dataset	Control			Affected		
			FC	nominal p	FDR p	FC	nominal p	FDR p
**NEUROD6**	10025910121	GSE44772	-1.2362847	**3.02E-07**	**1.39E-05**	-1.1323946	**0.00015085**	**0.03473297**
	220045_at	GSE1297	1.39363797	0.10085121	0.25601135	-1.0475343	0.68696581	0.99991996
	220045_at	GSE5281	-1.0145448	0.93446514	0.96263555	1.25633605	0.14590227	0.99993374
	8138882	GSE36980	1.06744845	0.76937498	0.95542564	1.11144682	0.7256297	0.92045354
	GI_27475984-S	GSE15222	-1.2024455	**0.03043733**	0.372892	-1.0654889	0.49360351	0.65785265
**SNAP25**	10025909548	GSE44772	-1.1537545	**1.45E-05**	**0.00016149**	-1.0914513	**0.0070681**	0.111054
	10023826155	GSE44772	-1.207256	**1.21E-05**	**0.00014075**	-1.1256482	**0.00470476**	*0.09943619*
	202507_s_at	GSE1297	3.58759616	**0.00101249**	**0.02813126**	-1.0073225	0.98787458	0.99991996
	202507_s_at	GSE5281	2.17223	0.10669549	0.3116042	1.41493808	0.36283777	0.99993374
	202508_s_at	GSE1297	1.45276767	**0.0137622**	0.09022159	1.68695618	0.31467102	0.99991996
	202508_s_at	GSE5281	1.66005764	*0.07758243*	0.28036503	1.20484363	0.51533723	0.99993374
	1556629_a_at	GSE1297	-			-		
	1556629_a_at	GSE5281	-1.3977231	*0.07965275*	0.28262795	1.24211177	0.47687631	0.99993374
	8060963	GSE36980	-1.0560367	0.50499275	0.88075331	-1.5379373	**0.02616377**	0.42854765
	GI_18765734-A	GSE15222	1.16812504	0.20276032	0.69523356	-1.5131451	**0.01037134**	*0.07341329*

(Largest 2 datasets by sample size denoted by underlining)
Each probe available on each platform for these genes was included.

Supplementary Table 12. Fisher's exact test for detecting gender bias within each dataset.

For four out of five gene expression datasets, no significant interaction was detected between gender and control or disease status. For one dataset (GSE44772) a significant interaction was detected with $p < 0.0002$, with a greater number of males within controls (reflecting the overall population structure in this disease).

Dataset	p value (Fisher's exact test)
GSE1297	0.35
GSE5281	0.10
GSE15222	0.46
GSE36980	1
GSE44772	0.0002
